# Evaluating the effect of measles and rubella mass vaccination campaigns on seroprevalence in India: a before-and-after cross-sectional household serosurvey in four districts, 2018–2020

**DOI:** 10.1016/S2214-109X(22)00379-5

**Published:** 2022-10-11

**Authors:** Manoj V Murhekar, Nivedita Gupta, Alvira Z Hasan, Muthusamy Santhosh Kumar, V Saravana Kumar, Christine Prosperi, Gajanan N Sapkal, Jeromie Wesley Vivian Thangaraj, Ojas Kaduskar, Vaishali Bhatt, Gururaj Rao Deshpande, Ullas Padinjaremattathil Thankappan, Avi Kumar Bansal, Sanjay L Chauhan, Gangandeep Singh Grover, Arun Kumar Jain, Ragini N Kulkarni, Santanu Kumar Sharma, Itta K Chaaithanya, Sanchit Kharwal, Sunil K Mishra, Neha R Salvi, Sandeep Sharma, Nilanju P Sarmah, R Sabarinathan, Augustine Duraiswamy, D Sudha Rani, K Kanagasabai, Abhishek Lachyan, Poonam Gawali, Mitali Kapoor, Arpit Kumar Shrivastava, Saurabh Kumar Chonker, Bipin Tilekar, Babasaheb V Tandale, Mohammad Ahmad, Lucky Sangal, Amy Winter, Sanjay M Mehendale, William J Moss, Kyla Hayford

**Affiliations:** a Indian Council of Medical Research (ICMR)-National Institute of Epidemiology, Chennai, India; b Division of Epidemiology and Communicable Diseases, Indian Council of Medical Research, New Delhi, India; c International Vaccine Access Center, Department of International Health, Johns Hopkins Bloomberg School of Public Health, Baltimore, MD, USA; d Department of Epidemiology and Statistics, University of Georgia, Athens, GA, USA; e Department of Epidemiology, Johns Hopkins Bloomberg School of Public Health, Baltimore, MD, USA; f ICMR-National Institute of Virology, Pune, India; g ICMR-National JALMA Institute for Leprosy & Other Mycobacterial Diseases, Agra, India; h ICMR- National Institute for Research in Reproductive and Child Health, Mumbai, India; i Directorate of Health Services, Government of Punjab, Chandigarh, India; j ICMR-National Institute of Pathology, New Delhi, India; k ICMR-Regional Medical Research Centre, NE Region, Dibrugarh, India; l Department of Health Research, Model Rural Health Research Unit-Dahanu, Maharashtra, India; m Department of Health Research, Model Rural Health Research Unit-Hoshiarpur, Punjab, India; n Department of Health Research, Model Rural Health Research Unit-Chabua, Assam, India; o WHO Country Office, New Delhi, India; p WHO, Southeast Asia Region Office, New Delhi, India; q PD Hinduja Hospital and Medical Research Centre, Mumbai, India

## Abstract

**Background:**

India did phased measles–rubella supplementary immunisation activities (MR-SIAs; ie, mass-immunisation campaigns) targeting children aged 9 months to less than 15 years. We estimated measles–rubella seroprevalence before and after the MR-SIAs to quantify the effect on population immunity and identify remaining immunity gaps.

**Methods:**

Between March 9, 2018 and March 19, 2020 we did community-based, cross-sectional serosurveys in four districts in India before and after MR-SIAs. 30 villages or wards were selected within each district, and one census enumeration block from each was selected as the survey cluster. Households were enumerated and 13 children in the younger age group (9 months to <5 years) and 13 children in the older ager group (5 to <15 years) were randomly selected by use of computer-generated random numbers. Serum samples were tested for IgG antibodies to measles and rubella viruses by enzyme immunoassay.

**Findings:**

Specimens were collected from 2570 children before the MR-SIA and from 2619 children afterwards. The weighted MR-SIA coverage ranged from 73·7% to 90·5% in younger children and from 73·6% to 93·6% in older children. Before the MR-SIA, district-level measles seroprevalence was between 80·7% and 88·5% among younger children in all districts, and between 63·4% and 84·5% among older children. After the MR-SIA, measles seroprevalence among younger children increased to more than 90% (range 91·5 to 96·0) in all districts except Kanpur Nagar, in which it remained unchanged 80·4%. Among older children, measles seroprevalence increased to more than 90·0% (range 93·7% to 96·5%) in all districts except Hoshiarpur (88·7%). A significant increase in rubella seroprevalence was observed in all districts in both age groups, with the largest effect in Dibrugarh, where rubella seroprevalence increased from 10·6% to 96·5% among younger children.

**Interpretation:**

Measles–rubella seroprevalence increased substantially after the MR-SIAs but the serosurvey also identified remaining gaps in population immunity.

**Funding:**

The Bill & Melinda Gates Foundation and Indian Council of Medical Research.

## Introduction

Measles and rubella are highly transmissible vaccine-preventable viral diseases. Measles remains an important cause of child mortality globally. Of the estimated 7 549 000 measles cases reported in 2020, 33·8% were in southeast Asia. India had the second highest number of infants not receiving a first dose of measles-containing vaccine (MCV1).^1^ The public health importance of rubella is mainly because of the teratogenic potential of the virus. Modelling studies using seroprevalence data indicated that India accounts for 38% (40 000 of 105 000) of the global burden of congenital rubella syndrome cases.^2^

In 1985, MCV1 was introduced in the universal immunisation programme in India. MCV1 coverage in India increased from 42·2% in 1992 to 87·9% in 2021.^3^, ^4^ In 2010, the Indian Government introduced a second dose of measles-containing vaccine, with additional catch-up campaigns targeting children aged 9 months to 10 years in 14 states in which MCV1 coverage was less than 80%.^5^ To achieve the goal of elimination of measles, and rubella and congenital rubella syndrome by 2023, India did measles–rubella supplementary immunisation activities (MR-SIAs) using measles–rubella containing vaccine (MRCV) from 2017 to 2019, targeting approximately 410 million children aged 9 months to <15 years.^6^ Since rubella-containing vaccine was not part of the universal immunisation programme in India until 2016, nationwide MR-SIAs (rather than targeted approaches) were chosen as the strategy to rapidly increase population immunity. These MR-SIAs were completed in 26 of the 28 Indian states (ie, all except West Bengal and the National Capital Territory of Delhi).

**Research in context**

**Evidence before this study**

On August 17, 2021, we searched PubMed for articles published from database inception to August 17, 2021, for English language articles assessing, via serological surveys, the effects of measles–rubella vaccination campaigns on population immunity, using the search terms “seroprevalence” AND “campaign” AND (“measles” OR “rubella”). A study from 1998 evaluated the effect of the Australian Measles Control Campaign on population immunity using residual blood specimens from public and private diagnostic laboratories. A 2021 study done in Zambia assessed the effect of a measles–rubella vaccination campaign, using blood samples from different cross-sectional surveys, a national biorepository for the precampaign, and a community-based serosurvey for post-campaign. Five studies did serosurveys only after a campaign to estimate the amount of population immunity and to identify immunity gaps. To our knowledge, ours is the first study to use household serosurveys, following the same study design both before and after supplementary immunisation activities, to measure the effect of an immunisation campaign on population immunity.

**Added value of this study**

Our study adopted the key modifications of the revised WHO *Vaccination Coverage Cluster Survey Manual* (2018). Household serosurveys were done in four districts of India before and after measles–rubella supplementary immunisation activities (MR-SIAs), using a three-stage cluster design to ensure probability-based selection in all stages and centralised selection of study participants. Our study revealed that population immunity to measles and rubella increased after the MR-SIA, although the magnitude varied by age group and district. After the MR-SIA, rubella seroprevalence among both younger children (9 months to <5 years) and older children (5 years to <15 years) and measles seroprevalence among children aged 5 years to <15 years was significantly higher in all districts compared with pre-MR-SIA seroprevalence. Vaccination coverage of the MR-SIA was lower in all districts than the WHO target of at least 95%. Our study showed that post-MR-SIA seroprevalence estimates were higher than MR-SIA vaccination coverage in some districts. In Palghar and Dibrugarh, in which MR-SIA coverage was 90% or higher, measles and rubella seroprevalence was close to 95% after the MR-SIA. In Hoshiarpur, the rubella seroprevalence after the MR-SIA among children aged 5 to <15 years was higher than 95%. Both measles and rubella seroprevalence after the MR-SIA were lower among children aged 9 months to less than 5 years in Kanpur Nagar than in other districts. Our study highlighted the added value of serosurveys before and after the MR-SIA to show the effect of the vaccine, while documenting remaining immunity gaps in specific age groups and geographical locations.

**Implications of all the available evidence**

The increase in seroprevalence after the MR-SIA is expected to further reduce the transmission of measles and rubella viruses in India. However, strengthening case-based surveillance and sustaining high coverage of routine immunisation is necessary for India's progress towards measles and rubella elimination. These findings highlight the added value of serology in documenting the effect of MR-SIAs on population immunity and remaining immunity gaps.

Measles and rubella are acute-immunising and antigenically stable viral pathogens; hence, serology is a good marker of either past infection or vaccination. Although well conducted vaccine-coverage surveys provide surrogate data on population immunity, they do not capture immunity acquired from natural infection or the absence of protection in vaccinated people who do not mount an immune response. Serosurveys, by comparison, provide a composite picture of population immunity that is due to vaccination and natural infection.^7^ Furthermore, estimation of reliable vaccine coverage depends upon the availability of children's immunisation cards, which is generally lower among older children than among younger ones (mainly because cards can get lost over time). During the MR-SIAs in India, vaccination coverage was estimated using administrative methods, except in Karnataka and Tamil Nadu, in which coverage was independently evaluated. Serosurveys can provide age-specific seroprevalence estimates across wide age ranges, generating susceptibility profiles of communities in different risk settings.^7^

We did community-based serosurveys before and after the MR-SIAs to quantify the effect of the world's largest MR-SIA on population immunity and to estimate age-group specific seroprevalence against measles and rubella viruses after the MR-SIAs to identify remaining immunity gaps.

## Methods

### Study design and participants

Between March 9, 2018 and March 19, 2020 we did community-based, cross-sectional serosurveys before and after implementation of MR-SIAs in four districts of India: Hoshiarpur (Punjab), Palghar (Maharashtra), Kanpur Nagar (Uttar Pradesh), and Dibrugarh (Assam; appendix p 3). These districts were selected because of the presence of model rural health research units. These units were established to develop health-research infrastructure in remote rural areas and to promote the transfer of technology needed to improve the quality of health services for rural populations. The surveys were done among two age groups: from 9 months to under 5 years, and from 5 years to under 15 years, during 2018–2020 (for exact dates, see appendix p 4).

Following the guidance in the WHO *Vaccination Survey Manual*^8^ and ICF International, Disability and Health *Demographic Health Survey Sampling and Household Listing Manual*,^9^ we adopted a three-stage cluster design. First, we selected 30 clusters (ie, villages in rural areas and wards [the lowest administrative subdivision in urban area in India] in urban areas) from each district, using probability proportional to the population size based on data in the 2011 census. Second, one census enumeration block was randomly selected from each cluster by use of computer-generated random numbers. A census enumeration block is a well defined area in a village or ward, containing 120–150 households and up to 800 people allotted to an enumerator at the time of the census, which occurs once every 10 years.^10^ Third, all individuals in the census enumeration block were enumerated and 13 eligible individuals per age group were selected by simple random sampling by use of computer-generated random numbers. The surveys before and after the MR-SIA were done in separate clusters to reduce the potential influence of the survey on vaccination during the campaign (appendix p 5).

We estimated a sample size of 210 per age group in each district, assuming rubella seroprevalence of 50% among both the younger and older age groups, with an absolute precision of 10%, a design effect of 2, and a 95% confidence level. Published data about rubella seroprevalence among children from India before the MR-SIA were not available when we designed the study. With 30 clusters from each district, we required a minimum of seven individuals per age group per cluster. To account for non-participation owing to locked houses, unavailability, or refusals, we selected 13 individuals per age group per cluster and attempted to enrol all selected individuals.

After enumeration, data were uploaded to the server at the Indian Council of Medical Research National Institute of Epidemiology and 13 individuals per age group were randomly selected by use of computer-generated random numbers. In the surveys before the MR-SIA, individuals were selected on the basis of their age at the time of the survey, whereas in surveys after the MR-SIA, selection was based on age at the time of MR-SIA, to ensure that the children had been eligible to receive MR-SIA vaccination. The survey team visited all randomly selected individuals to collect information on sociodemographic characteristics and vaccination history, and a blood sample, after obtaining informed consent or assent. Written informed consent from parents of children aged between 9 months to <15 years, oral assent from children aged between 7 to <12 years, and written assent from children 12 to <15 years were obtained before participation in the survey. Up to three household visits were made to enrol selected individuals.

The Institutional Ethics Committees of Indian Council of Medical Research National Institute of Epidemiology, Chennai, India; Johns Hopkins Bloomberg School of Public Health, Baltimore, USA, and the individual study sites approved the protocol (https://nie.gov.in/images/imrvi/Revised-IMRVIProposal-7Aug2019.pdf).

### Procedures

A trained phlebotomist collected from each participant 2 mL of venous blood in a serum separator tube. Blood samples were kept at room temperature for 30 min, centrifuged at 3000 revolutions per min for 10 min, and stored at 4–8°C in cold boxes until they were transported to the model rural health research unit site laboratory, where serum samples were aliquoted and stored at –20°C. At the end of the survey, serum samples were transported to the Indian Council of Medical Research National Institute of Virology, Pune, under cold-chain conditions.

Serum samples were tested for IgG antibodies against measles and rubella viruses using commercial enzyme immunoassays (Euroimmun AG, Lübeck, Germany; product codes EI 2610–9601G for measles and EI 2590–9601G for rubella) following the manufacturer's instructions. Four samples per plate were randomly selected in duplicate by use of computer-generated random numbers to monitor intraplate variability. Samples with measles IgG of 200 mIU/mL or more were considered seropositive, less than 150 mIU/mL as seronegative, and from 150 to less than 200 mIU/mL as equivocal. The corresponding thresholds for rubella IgG were at least 11 mIU/mL for seropositive, less than 8 mIU/mL for seronegative, and from at least 8 to less than 11 mIU/mL for equivocal. Equivocal samples were retested in duplicate using the same assay. From the three qualitative results, we selected the most commonly observed one as the final result, with samples determined to be equivocal being treated as positive in the analyses. We also did a sensitivity analysis, treating equivocal results as negative.

Initial measles IgG antibody results from two serosurveys (in Dibrugarh and Hoshiarpur) after the MR-SIA had unexpectedly lower seroprevalence compared with the serosurveys before the MR-SIA. The manufacturer confirmed that there had been a change to one of the four kit-provided calibrators, starting with one lot from midway through the testing of the serosurveys after the MR-SIA. This change affected the slope and intercept of the standard curve used to convert optical density values to IgG antibody concentrations (mIU/mL). The change primarily affected specimens near to the equivocal threshold, resulting in lower quantitative values relative to specimens calculated with a standard curve using the prior calibrator. We tested 403 specimens, randomly selected by use of computer-generated random numbers, from the serosurveys in Dibrugarh and Hoshiarpur before the MR-SIAs using the lot with the changed calibrator and used the lot-to-lot linear relationship to derive a quantitative correction factor that was then applied to the Dibrugarh and Hoshiarpur post-MR-SIA specimens to allow comparability between the serosurveys before and after the MR-SIAs (appendix pp 6–10). No changes were made to other serosurvey results, as we attributed the lower results to the calibrator change, which only affected the two serosurveys in Dibrugarh and Hoshiarpur.

### Statistical analysis

We described the sociodemographic characteristics of the participants enrolled during the serosurveys done before and after the MR-SIA. We classified residences as rural, urban slum, and urban non-slum, as per census definitions (appendix p 5). Vaccination coverage estimates were based on the number of vaccine doses received according to the vaccination card or, if the card was unavailable, the recall of the mother or caregiver. Children with an unknown vaccination status based on recall were treated as not vaccinated. Age-specific weighted seroprevalence of IgG antibodies against measles and rubella viruses were estimated with 95% Wald CIs using a random-intercept logistic regression model that included sampling weights (appendix p 5). Penalised regression splines were used to estimate measles and rubella seropositivity by age (appendix p 5). In addition to the analysis prespecified in the protocol, we also estimated predicted measles seroprevalence on the basis of vaccination coverage, assuming a vaccine efficacy of 84% for one dose of measles vaccine, and of 97% for two or more doses,^11^ and compared this estimate with the observed seroprevalence. Using logistic regression analysis, we compared seronegative versus seropositive children to identify factors associated with seronegativity. This comparison was done separately for children in the younger and older age groups, by use of the results from before and after the MR-SIA. Variables with p<0·2 on univariate analysis were included in a multivariable model. Odds ratios (with 95% CIs) were adjusted for sex, religion, mother's education or mother's occupation, type of residence or house, type of toilet provision, number of vaccine doses received, measles–rubella campaign dose coverage, and district. The analyses were done using the survey data analysis module in STATA SE (version 13.0) and R (version 3.4.4).

### Role of the funding source

The Bill & Melinda Gates Foundation had no role in the study design, data collection, data analysis, data interpretation, or writing of the report. Investigators from Indian Council of Medical Research, New Delhi, were involved in the study design, coordination of the study, interpretation of data, writing of the report, and in the decision to submit for publication; they were not involved in data collection and analysis.

## Results

**Figure 1:**
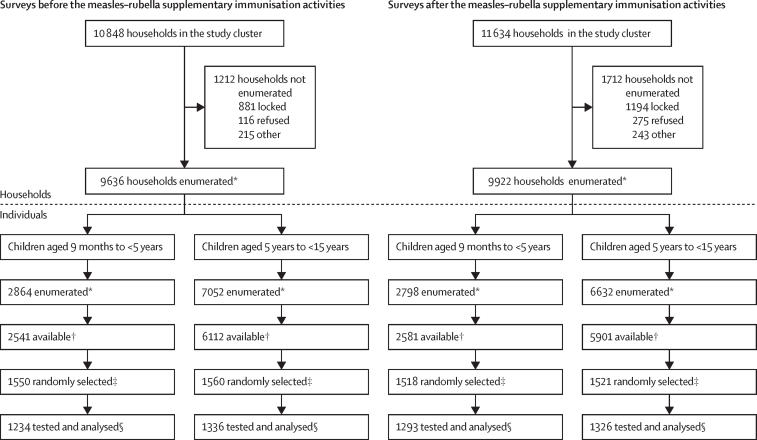
Study profile *Enumeration involved visiting all households in the cluster to collect identification details (name, date of birth or age, and gender). †Children available for the next 3 days. ‡Automated random selection of children from enumeration data using an in-house app developed for the purpose. §Children with adequate sample volume were tested and analysed in the final dataset.

During the surveys before the MR-SIA, study teams enumerated 9636 (88·8%; range: 83·6–92·8) of the 10 848 households from 120 clusters selected from four districts. Blood samples were collected from 1234 (79·6%; range 72·1–83·8) of the 1550 randomly selected children in the younger age group and 1336 (85·6%; range 77·2–90·3) of 1560 randomly selected children in the older age group (figure 1).

In the surveys after the MR-SIA, study teams enumerated 9922 (85·3%; range 78·8–88·2) of 11 634 households from 117 clusters within the four districts (three clusters in Dibrugarh could not be surveyed owing to the COVID-19 pandemic). Blood samples were collected from 1293 (85·2%; range 81·8–88·6) of 1518 randomly selected children in the younger age group and 1326 (87·2%; range 82·3–92·6) of 1521 randomly selected children in the older age group (figure 1). Enrolment of study participants by district is shown in the appendix (appendix pp 11–14).

Among the younger children included in the surveys before and after the MR-SIA, 1270 (50·3%) of 2527 were female, 2163 (86·4%) of 2504 were Hindus, and 1673 (67·5%) of 2479 were of general caste or other backward class (OBC; table 1). Among the older children who were included, 1279 (48·0%) of 2662 were female, 2252 (85·2%) of 2644 were Hindus, and 1760 (67·2%) of 2620 were of general caste or OBC (table 1). The distribution of children in the surveys before and after the MR-SIA within the two age groups and four districts were similar by gender. However, some characteristics (eg, the mother's occupation, education, residence, type of house, type of toilet, and health-seeking behaviour for her child's vaccination) differed significantly between surveys done before and after the MR-SIA, and across districts (appendix pp 15–18).

**Table 1:** Participant characteristics

	**Age 9 months to <5 years**		**Age 5 to <15 years**	
	Pre-SIA survey (n=1234)	Post-SIA survey (n=1293)	Pre-SIA survey (n=1336)	Post-SIA survey ((n=1326)
**Sex**				
Female	636/1234 (52%)	634/1293 (49%)	638/1336 (48%)	641/1326 (48%)
Male	598/1234 (48%)	659/1293 (51%)	698/1336 (52%)	685/1326 (52%)
**Religion**				
Hindu	1048/1215 (86%)	1115/1289 (87%)	1133/1324 (86%)	1119/1320 (85%)
Muslim or Christian	82/1215 (7%)	66/1289 (5%)	84/1324 (6%)	83/1320 (6%)
Sikh, Buddhist, or Jain	85/1215 (7%)	108/1289 (8%)	107/1324 (8%)	118/1320 (9%)
Caste*				
General category	288/1203 (24%)	553/1276 (43%)	328/1312 (25%)	568/1308 (43%)
Other backward classes	493/1203 (41%)	339/1276 (27%)	518/1312 (39%)	346/1308 (27%)
Scheduled caste	250/1203 (21%)	216/1276 (17%)	286/1312 (22%)	227/1308 (17%)
Scheduled tribe	172/1203 (14%)	168/1276 (13%)	180/1312 (14%)	167/1308 (13%)
**Mother's occupation**				
Employed	162/1230 (13%)	271/1285 (21%)	242/1308 (19%)	285/1297 (22%)
Homemaker	1068/1230 (87%)	1014/1285 (79%)	1066/1308 (81%)	1012/1297 (78%)
**Mother's level of education**				
Graduate or higher	148/1228 (12%)	220/1287 (17%)	132/1308 (10%)	131/1297 (10%)
11–12 years (higher secondary school)	128/1228 (10%)	181/1287 (14%)	111/1308 (8%)	146/1297 (11%)
6–10 years (middle or high school)	538/1228 (44%)	503/1287 (39%)	526/1308 (40%)	520/1297 (40%)
1–5 years (primary school)	172/1228 (14%)	211/1287 (16%)	212/1308 (16%)	266/1297 (21%)
Illiterate	242/1228 (20%)	172/1287 (13%)	327/1308 (25%)	234/1297 (18%)
**Type of residence**				
Rural	78/1234 (64%)	741/1293 (57%)	819/1336 (61%)	761/1326 (57%)
Urban slum	168/1234 (14%)	237/1293 (18%)	202/1336 (15%)	241/1326 (18%)
Urban non-slum	283/1234 (23%)	315/1293 (24%)	315/1336 (24%)	324/1326 (24%)
**Type of house**†				
Kutcha	252/1217 (21%)	343/1289 (27%)	281/1324 (21%)	302/1320 (23%)
Semi-pucca	359/1217 (29%)	282/1289 (22%)	382/1324 (29%)	320/1320 (24%)
Pucca	606/1217 (50%)	664/1289 (52%)	661/1324 (50%)	698/1320 (53%)
**Toilet provision**				
Own toilet	944/1217 (78%)	1094/1289 (85%)	1057/1324 (80%)	1169/1320 (89%)
Shared or public toilet	102/1217 (8%)	104/1289 (8%)	99/1324 (7%)	76/1320 (6%)
No facilities or uses open space	171/1217 (14%)	91/1289 (7%)	168/1324 (13%)	75/1320 (6%)
**Health-seeking behaviour for vaccination**				
Public sector	1079/1215 (89%)	1193/1281 (93%)	1142/1322 (86%)	1236/1313 (94%)
Private or non-public sector	136/1215 (11%)	88/1281 (7%)	180/1322 (14%)	77/1313 (6%)

Data are n/N (%). Percentages do not all add up to 100% owing to rounding. SIA=supplementary immunisation activities.

* The Indian population was socially stratified into four groups based on caste.

† Kutcha houses are made from mud, thatch, or other low-quality materials; semi-pucca houses have high-quality walls with thatched roofs; pucca houses are made with high-quality materials throughout, including the floor, roof, and exterior walls.

Routine immunisation card availability ranged between 196 (63·0%) of 311 (in Kanpur Nagar) and 259 (80·7%) of 321 (in Hoshiarpur) during the surveys before the MR-SIA. A similar pattern was observed in surveys after the MR-SIA. In the the surveys before the MR-SIA, the weighted proportion of younger children who had received no MCV dose, based on their vaccination card or mother's recall, ranged between 9·5% [95% CI 3·8–15·2]; in Dibrugarh) and 27·4% [18·9–35·9] in Kanpur Nagar). By contrast, in the surveys after the MR-SIA, the proportion of younger children who had received no MCV dose ranged between 1·1% (95% CI 0·0–2·6; in Palghar) and 6·2% (2·9–9·5; in Kanpur Nagar). MR-SIA card availability among both younger and older children was lowest in Kanpur Nagar and highest in Palghar. The weighted coverage of MR-SIAs was between 73·7% (95% CI 65·3–82·2; in Kanpur Nagar) and 90·5% (85·5–95·5; in Dibrugarh) among younger children, and in older children was between 73·6% (64·5–82·8; in Kanpur Nagar) and 93·6% (90·1–97·0; in Palghar; table 2).

**Table 2:** Weighted coverage of vaccines containing measles or measles–rubella before and after the MR-SIA survey in India, 2018–20

	**Hoshiarpur, Punjab**		**Dibrugarh, Assam**		**Palghar, Maharashtra**		**Kanpur Nagar, Uttar Pradesh**	
	Survey before the MR-SIA	Survey after the MR-SIA	Survey before the MR-SIA	Survey after the MR-SIA	Survey before the MR-SIA	Survey after the MR-SIA	Survey before the MR-SIA	Survey after the MR-SIA
**Routine immunisation card availability**								
Age 9 months to <5 years	259/321 (81%)	255/339 (75%)	246/326 (75%)	232/311 (75%)	179/276 (65%)	216/324 (67%)	196/311 (63%)	162/319 (51%)
**MR-SIA card availability**								
Age 9 months to <5 years	NA	207/339 (61%)	NA	200/311 (64%)	NA	245/324 (76%)	NA	156/319 (49%)
Age 5 years to <15 years	NA	205/345 (59%)	NA	217/325 (67%)	NA	241/321 (75%)	NA	170/335 (51%)
Total coverage of doses of measles-containing vaccine among children aged 9 months to <5 years*								
0	11·0 (6·9–15·1)	4·2 (0·9–7·5)	9·5 (3·8–15·2)	1·2 (0·0–2·4)	19·9 (12·4–27·4)	1·1 (0·0–2·6)	27·4 (18·9–35·9)	6·2 (2·9–9·5)
1	22·3 (15·8–28·9)	10·9 (6·5–15·4)	41·2 (29·0–53·5)	27·6 (20·6–34·6)	21·9 (15·9–27·9)	8·1 (3·3–13·0)	24·5 (18·5–30·6)	10·4 (5·4–15·3)
2	66·7 (59·5–73·8)	28·3 (20·2–36·3)	49·2 (37·8–60·7)	22·2 (16·8–27·6)	58·2 (50·4–65·9)	19·6 (14·6–24·6)	48·1 (40·6–55·5)	24·1 (17·6–30·5)
3	NA	56·6 (48·8–64·4)	NA	49·0 (42·6–55·5)	NA	71·1 (64·8–77·4)	NA	59·3 (48·1–70·6)
Coverage of MR-SIA*								
Age 9 months to <5 years	NA	82·9 (75·8–90·0)	NA	90·5 (85·5–95·5)	NA	89·8 (86·0–93·7)	NA	73·7 (65·3–82·2)
Age 5 years to <15 years	NA	85·4 (80·7–90·0)	NA	90·0 (83·4–96·6)	NA	93·6 (90·1–97·0)	NA	73·6 (64·5–82·8)

Data are n/N (%) or % (95% CI). MR-SIA=measles–rubella supplementary immunisation activity. NA=not applicable.

* Data are weighted estimates for the coverage of measles-containing vaccine doses received via routine immunisation or MR-SIA according to the immunisation card or mother's recall.

**Figure 2:**
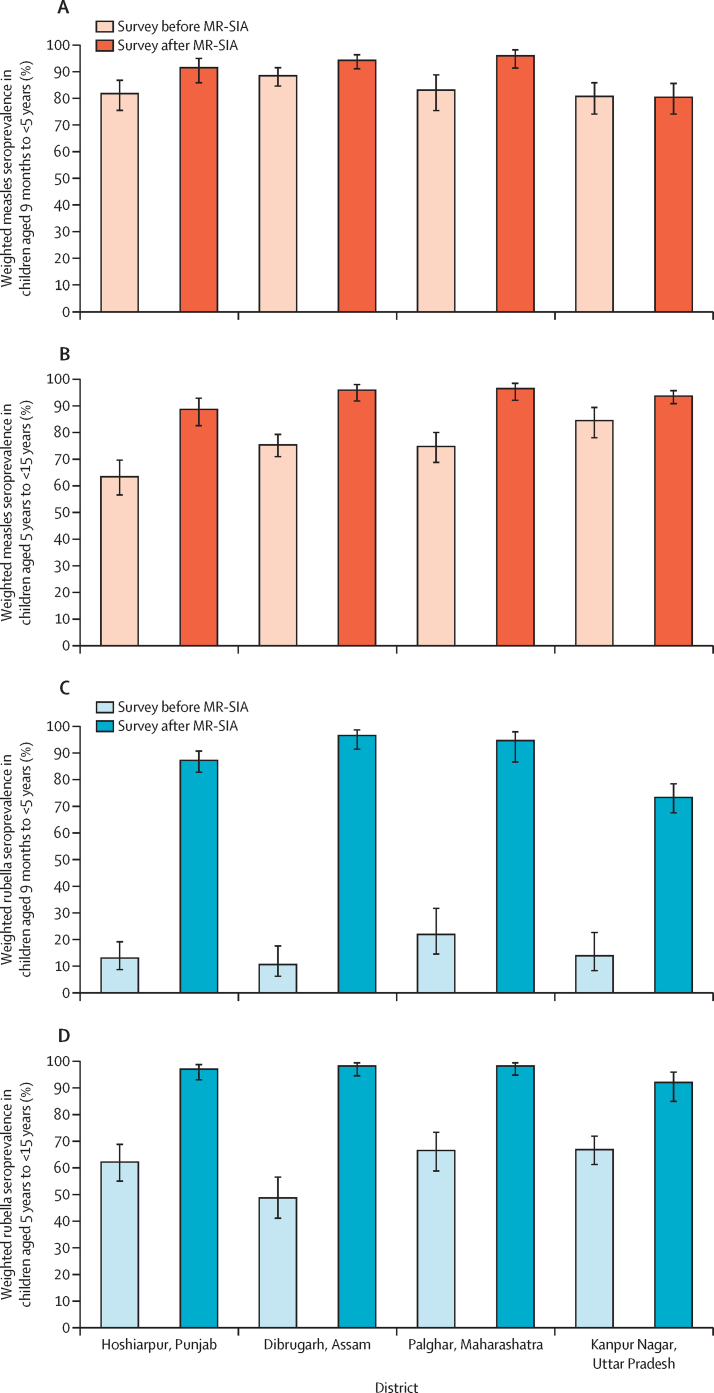
Seroprevalence of IgG antibodies against measles and rubella viruses before and after the MR-SIA, by district Bars represent the weighted seroprevalence estimates for the pre-MR-SIA serosurvey and the post-MR-SIA serosurvey. Lines at the top of each bar represent 95% CIs. Age-specific weighted seroprevalence of IgG antibodies against measles and rubella viruses were estimated with Wald 95% CIs (black bars) using a random-intercept logistic regression model that included sampling weights based on survey design. Equivocal results were classified as seropositive. SIA=supplementary immunisation activities.

Among the younger age group, post-SIA measles seroprevalence increased compared with pre-SIA seroprevalence in three districts (in Hoshiarpur from 81·8% [95% CI 75·5–86·8] to 91·5% [85·9–95·0]; in Dibrugarh from 88·5% [84·6–91·5] to 94·3% [91·1–96·4]; and in Palghar from 83·1% [75·4–88·8] to 96·0% [91·4–98·2]). However, in Kanpur Nagar, measles seroprevalence did not change between the pre-SIA (80·7% [95% CI 74·1–85·9]) and post-SIA serosurveys (80·4% [74·1–85·6]; figure 2A). Seroprevalence increased with the number of MCV doses received among younger children in the surveys both before and after the MR-SIA (appendix p 20).

Among older children, measles seroprevalence after the MR-SIA increased in all districts: in Hoshiarpur from 63·4% (95% CI 56·6–69·7) to 88·7% (82·6–92·9); in Dibrugarh from 75·4% (71·0–79·3) to 95·9% (91·9–98·0); in Palghar from 74·8% (68·8–80·0) to 96·5% (92·1–98·5); and in Kanpur Nagar from 84·5% (78·1–89·4) to 93·7% (90·9–95·7; figure 2B).

In both age groups, seroprevalence after the MR-SIA was similar among boys and girls and in rural, urban, and urban non-slum areas in all districts (appendix pp 19, 21).

A significant increase in rubella seroprevalence after the MR-SIA was observed in all districts among younger children (in Hoshiarpur from 13·0% [95% CI 8·7–19·1] to 87·2% [82·7–90·7]; in Dibrugarh from 10·6% [6·2–17·6] to 96·5% [91·4–98·6]; in Palghar from 21·9% [14·5–31·7] to 94·6% [86·6–97·9]; and in Kanpur Nagar from 13·9% [8·3–22·6] to 73·3% [67·5–78·4]; figure 2C; appendix p 22) and among older children (in Hoshiarpur from 62·2% [95% CI 55·0–68·8%] to 97·0% [93·0–98·7%]; in Dibrugarh from 48·7% [41·1–56·5%] to 98·2% [94·5–99·4%]; in Palghar from 66·5% [58·8–73·3%] to 98·2% [94·8–99·4%]; and in Kanpur Nagar from 66·8% [61·2–71·9%] to 92·0% [84·9–95·9]; figure 2D; appendix p 23). The proportion of samples with equivocal results and the estimated measles and rubella seroprevalence when classifying equivocal results as seronegative are presented in the appendix (pp 24–25).

**Figure 3:**
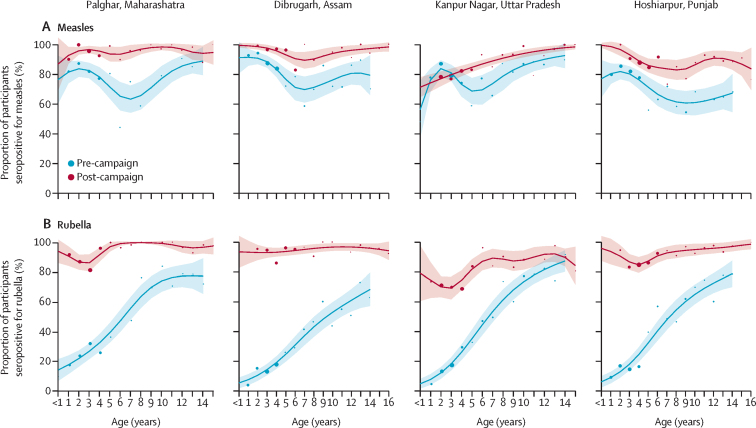
Age-specific measles and rubella seroprevalence among serosurvey participants, by district Weighted age-specific seroprevalence for measles (A) and rubella IgG (B), before and after the measles and rubella supplementary immunisation activities campaign. Shaded areas denote 95% CIs. Equivocal results were classified as positive. Coloured dots denote seroprevalence point estimates for each age by year, estimated using used semiparametric models with penalised regression smoothers, with dot size based on the number of participants who had available data. Age reflects participant age at time of the survey; owing to the time difference between the MR-SIA and the surveys, there were children enrolled in the post-MR-SIA surveys who were older than 15 years at time of the survey, including a small number aged 16 years or older in Dibrugarh and Hoshiarpur districts.

After the MR-SIAs, measles seroprevalence increased in all ages in Palghar, Dibrugarh, and Hoshiarpur. In Kanpur Nagar, an increase in post-MR-SIA measles seroprevalence was observed mainly among children aged 5–10 years. Before the SIAs, the lowest measles seroprevalence was observed among children aged 5–10 years in all districts; this immunity gap appeared to be filled after the MR-SIAs, most notably in Palghar (figure 3A).

The pre-MR-SIA rubella seroprevalence increased by age, reflecting cumulative exposure to rubella virus with increasing age. Following the MR-SIAs, rubella seroprevalence increased across all ages in all districts. However, rubella seroprevalence was lower among younger children in Kanpur Nagar than in the other districts (figure 3B).

Among younger children, the odds of being seronegative for measles were higher for children with zero (adjusted [aOR] 4·45 [95% CI: 2·00–9·87]) or one dose (aOR 2·97 [1·71–5·15]) of MCV compared with those who received three doses. Children who lived in a household with no toilet facilities or used open space for defecation (a proxy for socioeconomic status) had higher odds (aOR 3·10 [95% CI 1·57–6·11]) of being seronegative for measles. Children who did not receive a MR-SIA dose had higher odds (aOR 6·77 [95% CI 4·52–10·14]) of being seronegative for rubella. There were differences by districts in the odds for being seronegative for measles and rubella (appendix pp 26–27). Older children who did not receive a MR-SIA dose were more likely to be seronegative for measles (aOR 2·74 [95% CI 1·66–4·52]) or rubella (aOR 10·7 [6·21–18·3]) than those who received a MR-SIA dose (appendix pp 28–29). The factors associated with measles or rubella seronegativity before the MR-SIA are provided in the appendix (pp 30–33).

## Discussion

Globally, India's MR-SIA was one of the largest vaccination campaigns ever done. Given the scope, significance, and amount of investment, it is imperative to evaluate the necessity and effect of this historic SIA on population immunity. Our findings confirmed the existence of measles and rubella immunity gaps in both younger and older children before the MR-SIA and showed significant increases in population immunity to measles and rubella after the MR-SIA, although some immunity gaps remained. In the past, several studies documented the effect of SIAs using residual serum samples or by doing only post-SIA serosurveys.^12^, ^13^, ^14^, ^15^, ^16^, ^17^, ^18^ By contrast, we did community-based serosurveys, both before and after the MR-SIA, and each time used the same methods to estimate seroprevalence and document the effect of the MR-SIAs, thus reducing bias in our estimates.

Vaccination coverage data alone have been used to indirectly estimate vaccine derived immunity, but such estimates are limited by biases in vaccination coverage data (eg, missing vaccination cards) and unknown correlation between doses.^19^ Serological data directly estimate immunity that is due to vaccination and natural infection. Comparing seroprevalence estimates with estimates of vaccine-derived immunity across a range of dose-dependence assumptions for the study sites, seroprevalence was sometimes near the higher end of the vaccine-derived estimates, sometimes lower in the range, and at other times was outside the range (appendix p 36). This comparison highlights the value of serological data to correctly assess susceptibility and identify immunity gaps (eg, a measles immunity gap in children younger than 5 years in Kanpur Nagar) that would have otherwise been missed using vaccine coverage data alone. Serological data also provided estimates of immunity gaps in older children who did not have documentation of vaccine doses. Although the purpose of the paired serosurveys in this study was to quantify the effect of the MR-SIA and identify remaining immunity gaps, serosurveys can also inform targeted SIAs or routine vaccination strategies.^20^, ^21^ Further analyses into epidemiological, financial, and logistical trade-offs of targeted versus nationwide, non-selective SIAs are needed.

The potential of MR-SIAs to increase population immunity depends on precampaign population susceptibility and the correlation between routine and MR-SIA vaccine doses (ie, the probability that a vaccination dose risks being wasted on a child who has already received two-doses of vaccine and is seropositive). The lower the precampaign immunity and the lower the correlation between doses, the higher the effect of SIAs on population immunity.^22^ The pre-MR-SIA rubella seroprevalence was low (11–22%) among younger children as the rubella vaccine was not available in the public sector and the MR-SIAs resulted in a significant increase in post-MR-SIA rubella seroprevalence. Among younger children, the increase in measles seroprevalence was only marginal in Dibrugarh, Hoshiarpur, and Palghar. This scant increase could be explained by high pre-MR-SIA measles seroprevalence in all districts (range 81–89%) on account of routine immunisation and possibly natural infection. In Kanpur Nagar, measles seroprevalence did not increase, owing to the low (73%) MR-SIA coverage. Additional efforts to reach these remaining susceptible populations should be considered. Incomplete routine immunisation or non-receipt of an MR-SIA dose were significant risk factors for being seronegative for measles or rubella after the MR-SIA. Future SIAs using targeted approaches aimed at maximising impact and saving resources need to be guided by data on coverage of routine immunisation and previous SIAs, and case-based measles and rubella surveillance.

The WHO Global Measles and Rubella Strategic Plan^23^ set a milestone of achieving at least 95% coverage with MRCV during SIAs in every district. Although the reported coverage of MR-SIAs in the four surveyed districts ranged between 94% and 100%, the evaluated coverage was less than 95%. In India, the few coverage evaluation surveys done at district or sub-district level after the MR-SIA indicated coverage ranging between 68·8% in Imphal East (Manipur) to 90·5% in Kanchipuram (Tamil Nadu).^24^, ^25^, ^26^, ^27^ Large variations in MR-SIA coverage across districts highlight the need to document immunity gaps using both serological surveillance and strengthened district-level, case-based measles and rubella surveillance to guide immunisation strategies.

The MR-SIAs increased measles seroprevalence in all four districts and age groups, except for younger children in Kanpur Nagar. The increase in seroprevalence between serosurveys before and after the MR-SIA was not because of increases in the transmission of measles or rubella viruses. Measles and rubella surveillance data showed that no outbreaks were reported in the study districts between the pre-MR-SIA and post-MR-SIA serosurveys (WHO Country Office, India, unpublished). Despite this achievement, immunity gaps remain, and the extent of susceptibility was higher than that which was required to achieve measles elimination. A multicountry mathematical modelling study of measles serology data^28^ estimated that a threshold for contact-adjusted immunity of 93% is necessary to prevent measles outbreaks in European countries. Assuming similar age-assortative contacts in India, measles immunity gaps remain among older children in Hoshiarpur and Kanpur Nagar and among younger children in Kanpur Nagar. A mathematical modelling study evaluating rubella vaccine uptake scenarios in India^29^ estimated an 80% routine and campaign vaccination coverage to be sufficient to observe an annual and long-term reduction in congenital rubella syndrome cases across all Indian states. Our serosurvey identified rubella immunity gaps in Kanpur Nagar among children younger than 5 years that need to be filled before these birth cohorts reach reproductive age, to reduce any risk of rubella outbreaks and an increase in congenital rubella syndrome cases.

In future, measles and rubella serosurveys can be done in geographical areas with uncertain risk, such as those experiencing measles outbreaks despite high administrative vaccination coverage. Serosurveys can also identify age groups to be targeted by SIAs, particularly older individuals.^20^ In the context of measles and rubella elimination, estimated population immunity is a recommended key piece of evidence required for verification.^30^ Although community-based serosurveys are resource intensive, use of alternative methods such as dried blood spots during community-based surveys^31^ or residual serum samples from health facilities could substantially reduce the cost of serosurveys.

Our study has limitations. First, the study was done in four districts selected from the western, northern, eastern, and north-eastern regions of India. The findings might not reflect the actual effect of MR-SIAs in all districts in India. Second, our sample size might not be adequate to precisely estimate the rubella seroprevalence among younger children before the MR-SIA. However, our sample size was sufficient to estimate measles and rubella seroprevalence in other age groups. Third, although the same study design was used for the surveys before and after the MR-SIA, there were differences in some sociodemographic characteristics of the participants between the two surveys. Fourth, there was a loss of potential participants at different stages of sampling. Incomplete sampling frames and refusals could lead to random or systematic errors and affect the generalisability of the study findings. Fifth, the availability of routine and MR-SIA vaccination cards was only between 49·4% and 80·7% and coverage estimates based on recall might not be accurate.

In conclusion, measles–rubella seroprevalence increased substantially after the MR-SIAs but the serosurveys identified age groups and districts in which the extent of post-MR-SIA population immunity was lower. The increase in seroprevalence owing to the MR-SIA is expected to further reduce the transmission of measles and rubella viruses in India. However, continued case-based surveillance for measles and rubella and the maintenance of high coverage of measles and rubella vaccination through routine immunisation are necessary to track progress towards elimination and guide the immunisation programme.

## Data sharing

A subset of the key anonymised individual participant data collected during the study, along with a data dictionary, is available upon request made to the corresponding author, after approval of a proposal by the study core investigators with a signed data-access agreement.

## Declaration of interests

KH has been an employee by Pfizer Vaccines from October 26, 2020. All other authors declare no competing interests.
