# Supplementary material for: Evaluating the effect of measles and rubella mass vaccination campaigns on seroprevalence in India: a before-and-after cross-sectional household serosurvey in four districts, 2018–2020
Source: Lancet Glob Health. 2022 Oct 11;10(11):e1655–64. doi: 10.1016/S2214-109X(22)00379-5 (PMC9579355; doi:10.1016/S2214-109X(22)00379-5)
Supplement: Supplementary appendix [file mmc1.pdf]

THE LANCET
Global Health

Supplementary appendix

This appendix formed part of the original submission and has been peer reviewed. We post it as supplied by the authors.

Supplement to: Murhekar MV, Gupta N, Hasan AZ, et al. Evaluating the effect of measles and rubella mass vaccination campaigns on seroprevalence in India: a before-and-after cross-sectional household serosurvey in four districts, 2018–2020. *Lancet Glob Health* 2022; **10:** e1655–64.

SUPPLEMENTARY APPENDIX

SUPPLEMENT TO:

Evaluating the effect of measles and rubella mass vaccination campaigns on seroprevalence in India: a before-and-after cross-sectional household serosurvey in four districts, 2018–2020

Table of Contents **Page No.**

Supplementary methods

1. Figure 1: Map of India showing the districts selected for estimating seroprevalence of measles and rubella (MR) before and after MR supplementary Immunization Activities, 2018-2020……..………...3
2. Table 1: MRCV-SIA, Pre- and Post-SIA serosurvey dates by district ……...…………………….……….4
3. Supplementary methods……………………………………………………………………………….5
4. Measles IgG antibody quantitative adjustment………………………………………………….....6-10

Supplementary results

5. Figure 2: Flowchart describing the enrolment of participants in the pre and post-SIA serosurveys in Hoshiarpur, Punjab (A), Dibrugarh, Assam (B), Palghar, Maharashtra (C) and Kanpur Nagar, Uttar Pradesh (D) districts……………………………………………………………………….…..…11-14
6. Table 2: Characteristics of participants among children aged 9 months to <5 years, by district…...15-16
7. Table 3: Characteristics of participants among children aged 5 to <15 years by district……….…17-18
8. Table 4: Weighted seroprevalence (%) of IgG antibodies against measles before and after MR vaccine SIA by selected characteristics among children aged between 9 months and 5 years, 2018-20……..…19
9. Table 5: Seropositivity against measles by number of MCV doses received…………………..………20
10. Table 6: Weighted seroprevalence (%) of IgG antibodies against measles before and after MR vaccine SIA by selected characteristics among children aged 5 to <15 years, 2018-20…………………...……21
11. Table 7: Weighted seroprevalence (%) of IgG antibodies against rubella before and after MR vaccine SIA by selected characteristics among children aged 9 months to < 5 years, 2018-20………….........22
12. Table 8: Weighted seroprevalence (%) of IgG antibodies against rubella before and after MR vaccine SIA by selected characteristics among children aged 5 to <15 years, 2018-20………………………...23
13. Table 9: Number (%) of sera samples with equivocal results…………………………………………..24
14. Table 10: Overall seroprevalence (%) of IgG antibodies against measles and rubella before and after MR vaccine SIA among considering equivocal samples as test negative…………………………….25
15. Table 11: Factors associated with seronegative status against measles or rubella among children aged 9 months to <5 years in post-SIA survey, 2018-2020……………………………………………….26-27
16. Table 12: Factors associated with seronegative status against measles or rubella among children aged 5 years to <15 years in post-SIA survey, 2018-2020……………………………………………….28-29
17. Table 13: Factors associated with seronegative status against measles or rubella among children aged 9 months to <5 years in pre-SIA survey, 2018-2020………………………………………………….30-31
18. Table 14: Factors associated with seronegative status against measles or rubella among children aged 5 years to <15 years in pre-SIA survey, 2018-2020……………………………...……………32-33
19. Table 15: Summary of risk factors associated with measles and rubella seronegativity in pre and post SIA serosurveys……………………………………………………………………………………34
20. Table 16: Children age 12-23 months who have received measles vaccine (%) in the surveyed districts from National Family Health Surveys…………………………………………………………………35
21. Table 17: Comparison of predicted seroprevalence from the MCV coverage estimates and observed seroprevalence estimated during post-SIA serosurvey…………………………………………………36
22. STROBE Checklist……………………………………………………………………………..………37

1. **Figure 1: Map of India showing the districts selected for estimating seroprevalence of measles and rubella (MR) before and after MR supplementary Immunization Activities, 2018-20**

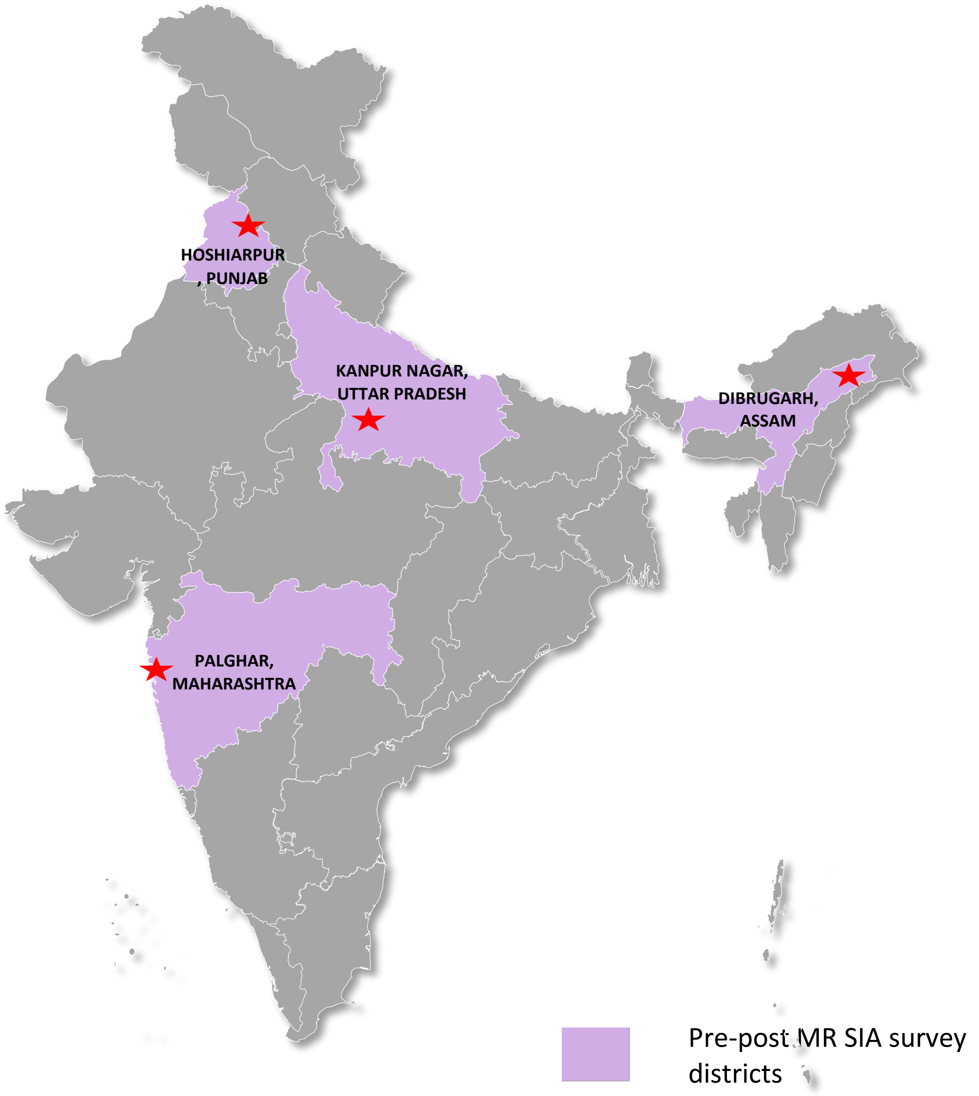

2. Table 1: Measles Rubella Supplementary Immunization Activity, Pre- and Post-SIA serosurvey dates by district

S.No.	Name of the district, state	MR SIA	Pre-SIA serosurvey	Post-SIA serosurvey	Interval between Pre-campaign survey and SIA (in months) [a]	Interval between SIA and post campaign survey (in months) [b]
1	Hoshiarpur, Punjab	May 1 – July 31, 2018	Mar 9 – Apr 16, 2018	Aug 31 – Dec 2, 2019	0.5-2	13-16
2	Dibrugarh, Assam	Aug 20 – Oct 31, 2018	Mar 23 – Apr 14, 2018	Oct 19, 2019 – March 19, 2020	4-5	12-14
3	Kanpur Nagar, Uttar Pradesh	Nov 26, 2018 – Feb 28, 2019	May 10 – Aug 10, 2018	Jun 1 – Sep 21, 2019	1-4	3-6
4	Palghar, Maharashtra	Nov 27, 2018 – Jan 28, 2019	Aug 6 – Sep 25, 2018	Apr 26 – Jun 19, 2019	2-4	3-5

[a]Approximate time between pre-SIA survey and campaign based on pre-SIA survey start date (minimum) and stop date (maximum) and the date of the campaign was started
[b]Approximate time between campaign and post-SIA survey based on approximate district-level campaign start date (minimum) and stop date (maximum) and the date the serosurvey was started.

3. Supplementary methods

Study procedures: The survey team first identified the CEB with the help of local health workers using the census maps procured from the regional census offices. Wherever census maps were not available or did not have clear landmarks, Anganwadi centers were used instead of CEBs. One Anganwadi center caters to a population of 300-800 to provide basic nutrition, health, and early education services. We listed the Anganwadi centers in the village/ward and randomly selected one Anganwadi center as an alternative to the CEB. After identifying the selected CEB and its boundaries, the team conducted a rapid mapping exercise to count the number of households and assessed if segmentation of the CEB was needed. A threshold of 70 households was set to ensure at least 13 individuals per age group were present in the study cluster. This threshold was based on birth rates, infant mortality ratio, and household size. If there were 70-140 households in the cluster, no segmentation was done. If there were more than 140 households, the cluster was segmented, and one segment was randomly selected by an independent statistician at ICMR-National Institute of Epidemiology, Chennai. We followed census definitions to classify the clusters as rural, urban slum and urban non-slums. **Urban**: a) Statutory town - All places with a municipality, corporation, cantonment board or notified town area committee, etc. b) Census town - All other places which satisfied the following criteria: (i)A minimum population of 5,000 (ii) At least 75 percent of the male main workers engaged in non-agricultural pursuits; and (iii) A density of population of at least 400 per sq. km. **Rural:** All areas which are not categorized as urban area are considered as rural area.[1] **Urban Slum:** (i) House located in specified areas in a town or city notified/recognized as slum by state or local government and union territory administration (or) (ii) House part of a compact area of at least 300 population or about 60-70 households of poorly built congested tenements, in an unhygienic environment with inadequate infrastructure and lacking in proper sanitary and drinking water facilities.[2] The survey team enumerated all individuals in the households in the study cluster. Identification details of all individuals who stayed in the house the previous night, including name, date of birth or age, gender, and availability for next 3 days, were collected using a tablet-based application.

Statistical Analysis: The sampling weight was calculated as the inverse of the product of selection probabilities of clusters (village or ward), segment, and individuals, corrected for non-response. Seroprevalence by sex and locality (rural/urban, non-slum and slum) was estimated using a similar model without weights. We did logistic regression to identify factors associated with seronegativity against measles or rubella after the MR-SIAs. Odds ratios (with 95%CI) were adjusted for sex, religion, mother's education or mother's occupation, type of residence or house, type of toilet provision, number of vaccine doses received, MR campaign dose coverage and district.

We used semiparametric models with penalized regression smoothers to estimate measles and rubella seropositivity by age. We selected the 'best' penalized regression spline between two spline basis functions (cubic regression spline, and thin plate regression spline) and two link functions (logit and complementary log-log), by minimizing Bayesian Information Criteria (BIC) for measles and rubella by district. The choice of the basis dimension was large enough to have enough degrees of freedom to represent the underlying data (Wood 2017)[5]. The 'mgcv' library in R was chosen to model the penalized regression splines for its computational efficiency, automated selection of the smoothness parameter, and goodness of fit to the data (Wood 2003, 2011 & 2017).[3-5] The final model selection for measles and rubella by district is given below.

Table. Final model selection for age-specific seroprevalence curves, regression and link functions

	Measles		Rubella	
District	Pre-campaign	Post-campaign	Pre-campaign	Post-campaign
Hoshiarpur, Punjab	CR, cloglog	CR, cloglog	TP, logit	CR, logit
Dibrugarh, Assam	TP, logit	CR, logit	CR, logit	TP, cloglog
Palghar, Maharashtra	CR, logit	CR, logit	CL, cloglog	TP, logit
Kanpur Nagar, Uttar Pradesh	CR, logit	TP, cloglog	CR, logit	CR, logit

Abbreviations: CR, cubic regression spline, TP, thin plate regression spline. All models used a smoothing parameter of 8 except Palghar District, Maharashtra, post-campaign rubella (smoothing parameter=6) and Hoshiarpur District, Punjab, post-campaign measles (smoothing parameter=7).

References

1.Census India. Rural and urban definition. : http://censusindia.gov.in/2011-prov-results/paper2/data_files/india/Rural_Urban_2011.pdf
2.Census India. Urban slum. https://pib.gov.in/newsite/PrintRelease.aspx?relid=68535
3.Wood SN (2003). "Thin-plate regression splines." Journal of the Royal Statistical Society (B), 65(1), 95-114.

4.Wood SN (2011). “Fast stable restricted maximum likelihood and marginal likelihood estimation of semiparametric generalized linear models.” Journal of the Royal Statistical Society (B), 73(1), 3-36.
5.Wood S (2017). Generalized Additive Models: An Introduction with R, 2 edition. Chapman and Hall/CRC.

4. Measles IgG Antibody Quantitative Adjustment

Initial measles IgG antibody results from the district-specific post-measles-rubella (MR) vaccination campaign serosurveys for the two districts tested in late 2019 and early 2020, indicated unexpectedly lower seroprevalence relative to the pre-campaign serosurveys (tested July through September 2018). These results were concerning and were discordant with estimated routine and campaign vaccination coverage in these districts. Although the measles IgG test kit remained the same (Euroimmun, EI 2610-9601 G), the kit lot differed between surveys, with approximately 8 different lots used prior to this time (E180312BR, E180332BR, E180508BS, E180905AG, E181204BF, E190204AG, E190321BI, E190409AD).

In reviewing the data on the four kit-provided calibrators, we observed an increase in the optical density (OD) value for the lowest calibrator (calibrator 4, set at 50 mIU/ml) starting with the lot midway (E190909RS) through the testing of the post-campaign specimens for the two districts (Figure 1). The calibrators are used to generate a point-to-point standard curve, where the slope and intercept of each line segment is used to convert the OD values to measles IgG antibody concentrations (mIU/ml) (Figure 2) from which to characterize a sample as seropositive, seronegative, or equivocal. Euroimmun confirmed a change to calibrator 4, starting with a lot midway through the testing of the post-campaign serosurveys, to achieve a more precise relationship to the WHO measles reference standard (personal communication). The reference OD value for this calibrator (as specified in the kit quality control inserts) increased from approximately 0.05 prior to the change to 0.15 after the change. Although the manufacturer reported this change had no qualitative impact using their validated threshold to define individual-level seropositivity ($\geq$ 275 mIU/ml), our analyses used the lower “immunity cut-off” described in the kit insert ($\geq$ 200 mIU/ml), which was based on the literature and recommended for assessing population-level seroprevalence. Their internal quality control investigations, which they referenced when reporting no qualitative impact of the change in calibrator, was limited to quality control specimens with a reference range < 75 IU/L and > 389 IU/L, excluding samples around the negative-equivocal threshold (200-275 mIU/ml per their validation). We observed this change to the calibrator qualitatively impacts results when using the lower threshold, making it difficult to compare seroprevalence results from the pre- and post-SIA serosurveys for the two districts where the calibrator changed between surveys.

4.A. Figure: Calibrator 4 (50 mIU/ml) optical density measurements across plates and kit lots. Each point represents a different plate (plate numbers restarted for each site tested so there may be multiple ‘Plate 1s’ in a figure). All plates were run at the National Institute of Virology (2018-2020). Lot E190909RS was the first lot where we observed the issue with the post-campaign samples testing lower than the pre-campaign. Additional lots were purchased to check for lot-specific issues, however similarly low findings were seen for additional lots. Higher calibrator 4 OD value were seen in all lots after E190909RS.

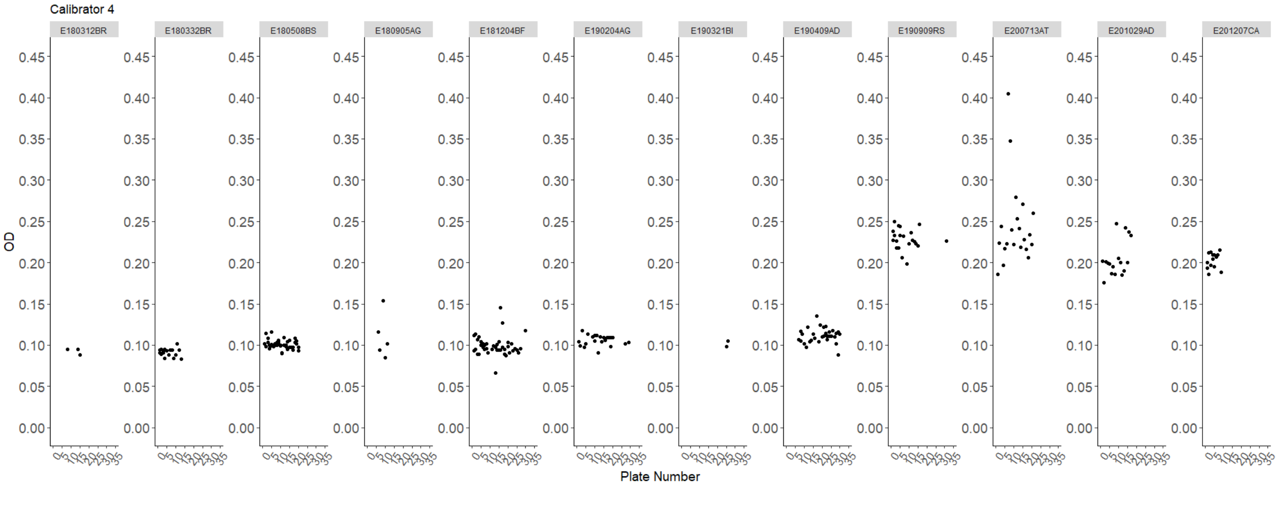

4.B. Figure. Example point-to-point standard curve used to convert OD values to concentration (mIU/ml). Calibrators from left to right points: calibrator 4 (50 mIU/ml); calibrator 3 (250 mIU/ml); calibrator 2 (1000 mIU/ml); calibrator 1 (5000 mIU/ml)

4.B.1. Calibrator 4 Optical Density 0.1 (observed with earlier lots)

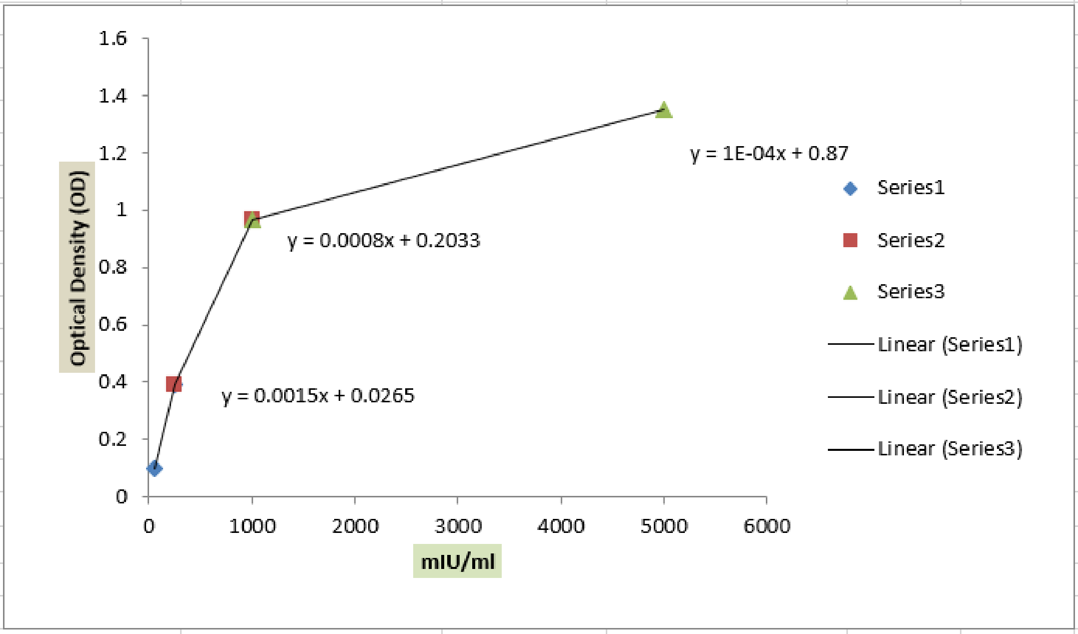

4.B.2: Calibrator 4 Optical Density 0.2 (observed with current lots; resulting in decreased slope and increased intercept for OD values falling within this line segment)

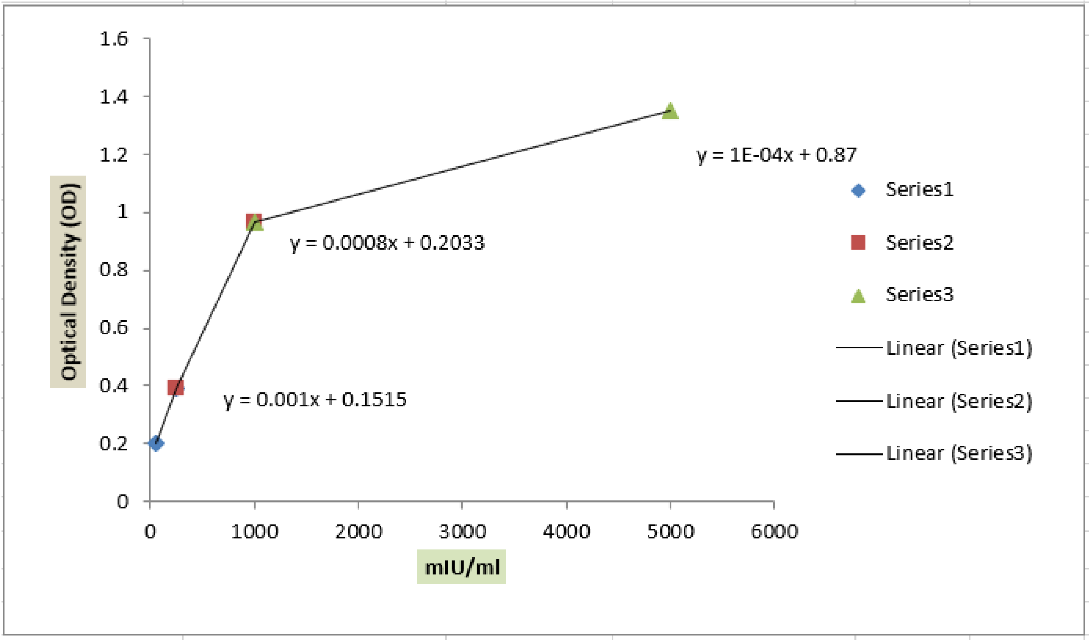

Two hundred specimens from post-campaign serosurveys tested on lots prior to the calibrator change were re-tested on a current lot (E201207CA), oversampling specimens near the thresholds (100 – 250 mIU/ml). These specimens were originally tested on three different lots (all prior to the calibrator change). Ten percent of the positive specimens and 48% of the equivocal specimens tested negative on the current lot (4.C.Table). Lower quantitative results were observed with the current lot (4.D.Figure) regardless of the lot used in the original testing. This evidence suggests the lots used to test the earlier post-campaign serosurveys behaved differently than the current lots and the change is likely attributed to the calibrator 4 change occurring in late 2019.

4.C. Table: Lot-to-lot qualitative comparison of post-campaign specimens from the Palghar District, Maharashtra and Kanpur Nagar District Uttar, Pradesh serosurveys. Red indicates qualitative change in the interpretation of results with the repeat lot (treating equivocal as positive).

Repeat measles qualitative results (E201207CA)	Original measles qualitative results (All lots)			
	**Positive**	**Equivocal**	**Negative**	**Total**
Positive	42	5	0	47
Equivocal	19	31	3	53
Negative	7	33	60	100
**Total**	68	69	63	200

4.D. Figure. Lot-to-lot quantitative comparison of post-campaign specimens from the Palghar District, Maharashtra and Kanpur Nagar District Uttar, Pradesh serosurveys

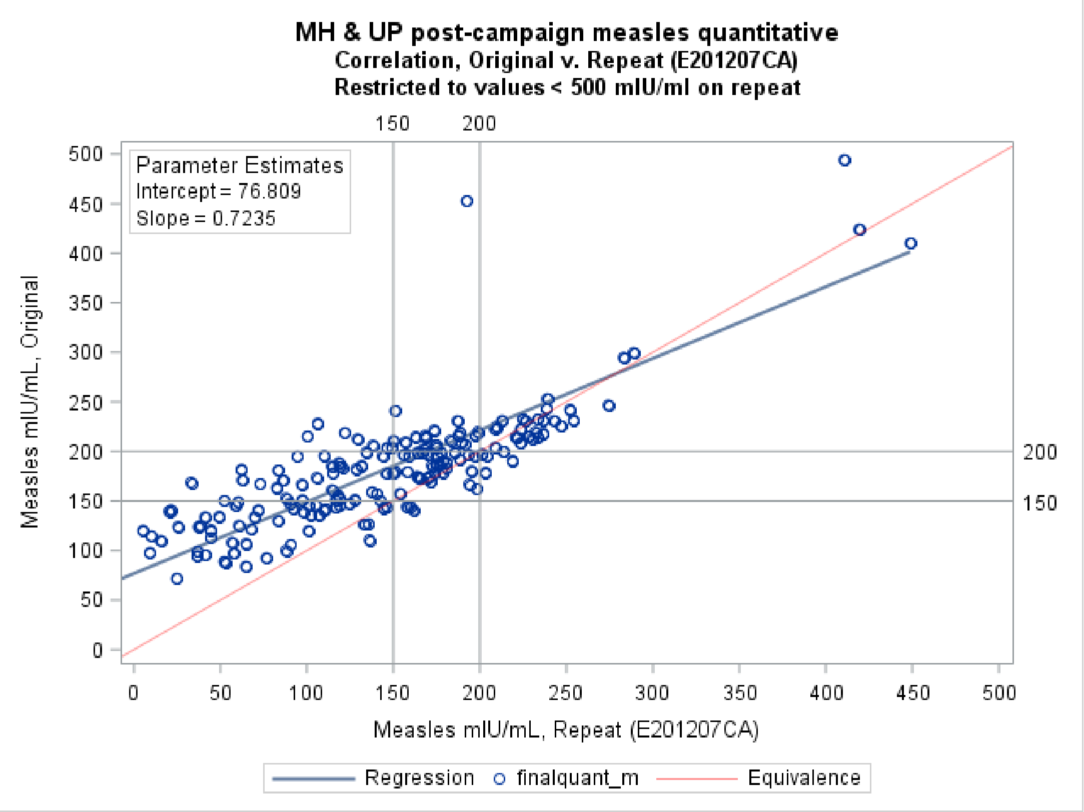

Since the change in calibrator 4 occurred between the pre-campaign and post-campaign serosurveys for two of the serosurveys (Hoshiarpur District, Punjab and Dibrugarh District, Assam), it is difficult to compare results and assess the impact of the campaign in these districts. To allow for comparability we tested four hundred and three specimens from the Punjab and Assam pre-campaign serosurveys, originally tested in 2018, using the current lot (E201207CA). The pre-campaign specimens were stratified based on measles quantitative values (low negative < 100 mIU/ml; high negative 100-199 mIU/ml; low positive 200-399 mIU/ml; and high positive > 400 mIU/ml) then specimens were randomly selected so that the bulk of samples selected were near the thresholds, with just a few high positives. Twenty percent of the positive specimens and 96% of the equivocal specimens tested negative on the current lot (4.E.Table). We were unable to do a similar exercise by testing post-campaign samples on an older lot as kits were available or expired.

4.E. Table: Lot-to-lot qualitative comparison of pre-campaign specimens from the Hoshiarpur District, Punjab and Dibrugarh District, Assam serosurveys. Red indicates qualitative change in the interpretation of results with the repeat lot (treating equivocal as positive).

**Repeat measles qualitative results (E201207CA)**	**Original measles qualitative results**			
	**Positive**	**Equivocal**	**Negative**	**Total**
Positive	127	0	0	127
Equivocal	21	4	0	25
Negative	37	94	120	251
**Total**	185	98	120	403

We used the lot-to-lot linear relationship for the Punjab and Assam pre-campaign specimens to develop a correction factor to apply to the Punjab and Assam post-campaign specimens (4.F. Figure). We chose to correct the post-SIA results using the earlier calibrator, rather than correct the pre-SIA using the updated calibrator, because we believe the post-campaign survey results run on the lots after the change in calibrator to be more problematic given the sensitivity issues we observed when comparing results for specimens run on prior lots. We saw a substantial increase in rubella seroprevalence after the SIA in these two districts, and in the absence of a rubella outbreak this change we attributed this change primarily to the SIA. While we cannot state which result is "correct", it was unrealistic to have a lower measles seroprevalence in the post-SIA survey relative to the pre-SIA survey in these districts, as we had observed prior to the correction, given the routine and SIA coverage and the rubella findings. Therefore, we decided to adjust the post-SIA specimens using the earlier calibrator.

We used 250 mIU/ml as the upper bound for developing the correction factor. This reflects the value of calibrator 3, which, along with the recently changed calibrator 4 (50 mIU/ml), form the line segment used to calculate concentrations for the lowest specimens (see example standard curve). Since the lot-to-lot relationship was similar between the two pre-campaign serosurveys and, given that we attributed this to a change in the calibrator rather than a lot-specific issue, we combined data from both pre-campaign serosurveys to determine the correction factor. The slope (1.3298) and intercept (85.7) from the lot-to-lot comparison was then applied to the Punjab and Assam post-campaign specimens that tested < 250 mIU/ml on the current lot.

4.F. Figure. Lot-to-lot quantitative comparison of post-campaign specimens from the Hoshiarpur District, Punjab and Dibrugarh District, Assam serosurveys

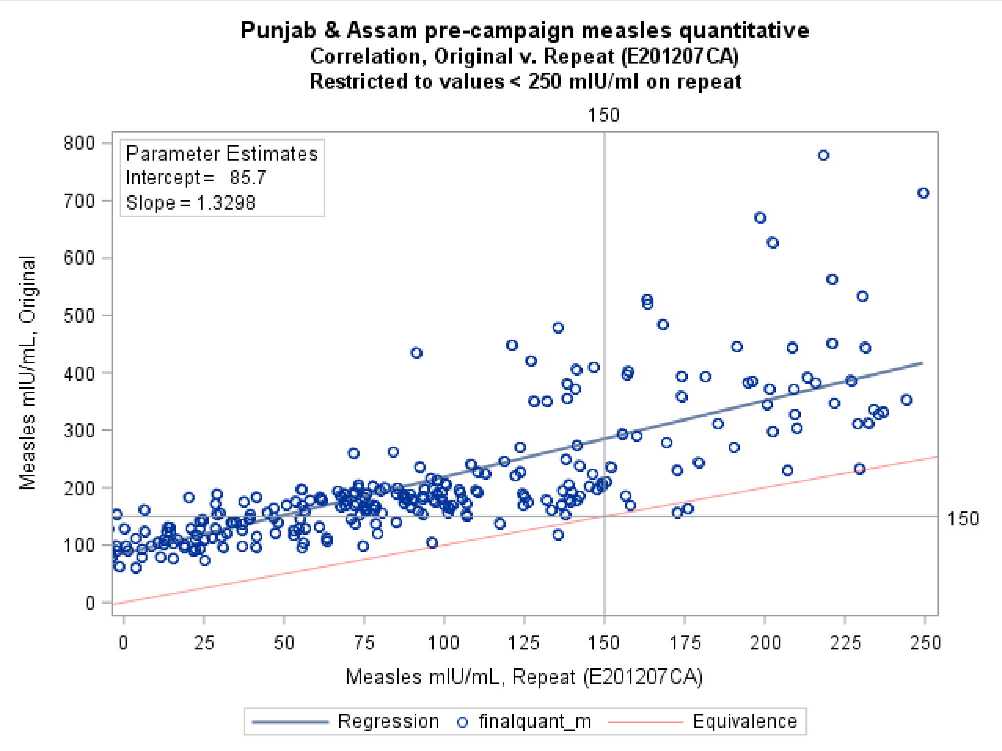

Based on the findings from the lot-to-lot comparison using specimens from other post-campaign serosurveys and the timing of the change to calibrator 4 we did not apply this adjustment to specimens from any other surveys.
Lot-to-lot testing of a sample of post-SIA specimens from the other two serosurveys also suggested the current kit behaved differently than the earlier kits and the observations were likely attributed to the change in the calibrator.

5. **Figure 2-A: Flowchart describing the enrolment of participants in the pre and post-SIA serosurveys by district**

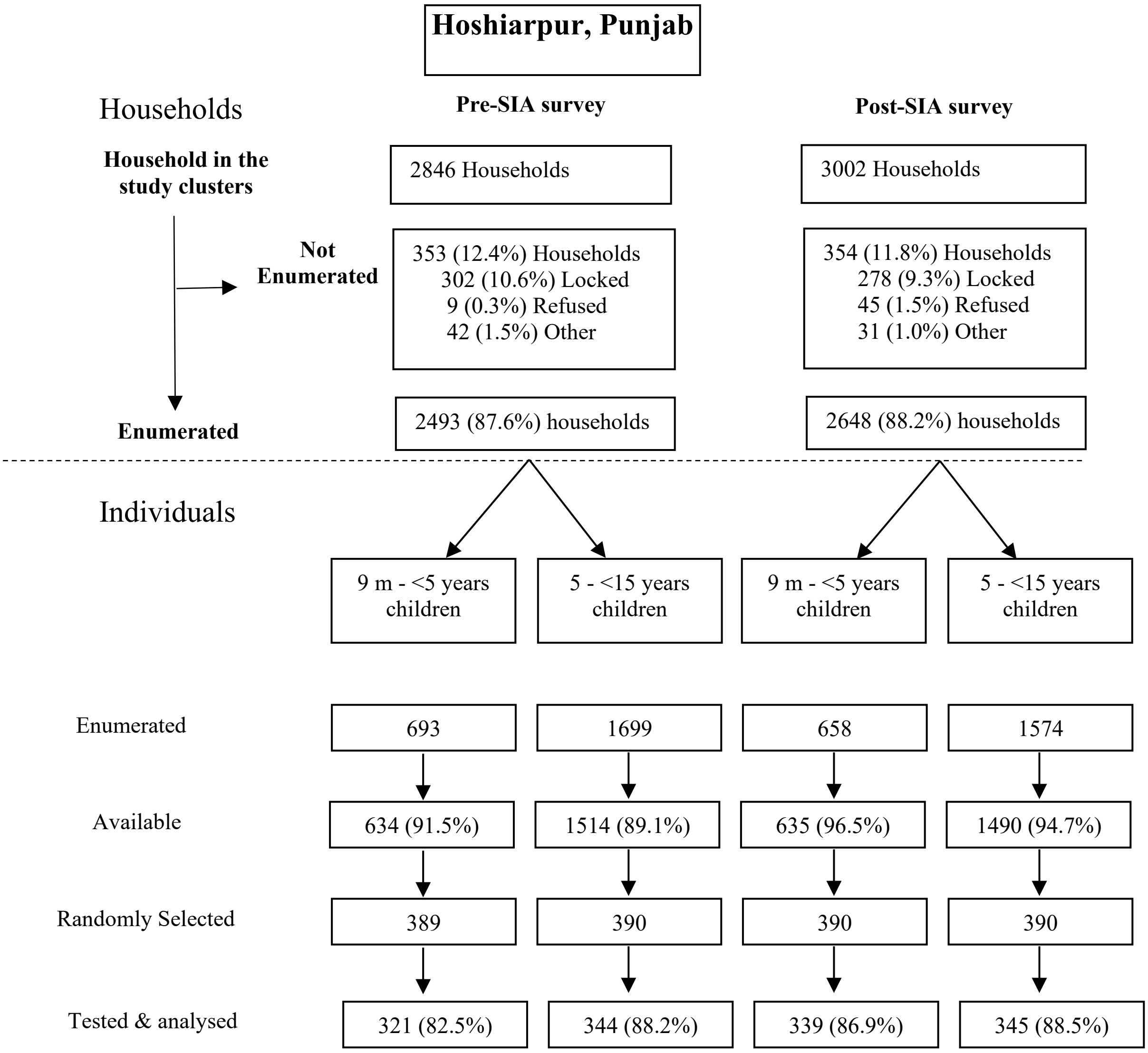

Enumerated: Visited all households in the cluster and collect identification details (name, date of birth/age, gender) **Available:** Children are available for the next three day**s Randomly selected:** Automated selection of children from enumeration data using android application. **Enrolled:** Data collected after obtaining consent/assent **Tested and analysed:** Children with adequate sample volume tested and analysed in the final dataset

Figure 2-B: Flowchart describing the enrolment of participants in the pre and post-SIA serosurveys by district

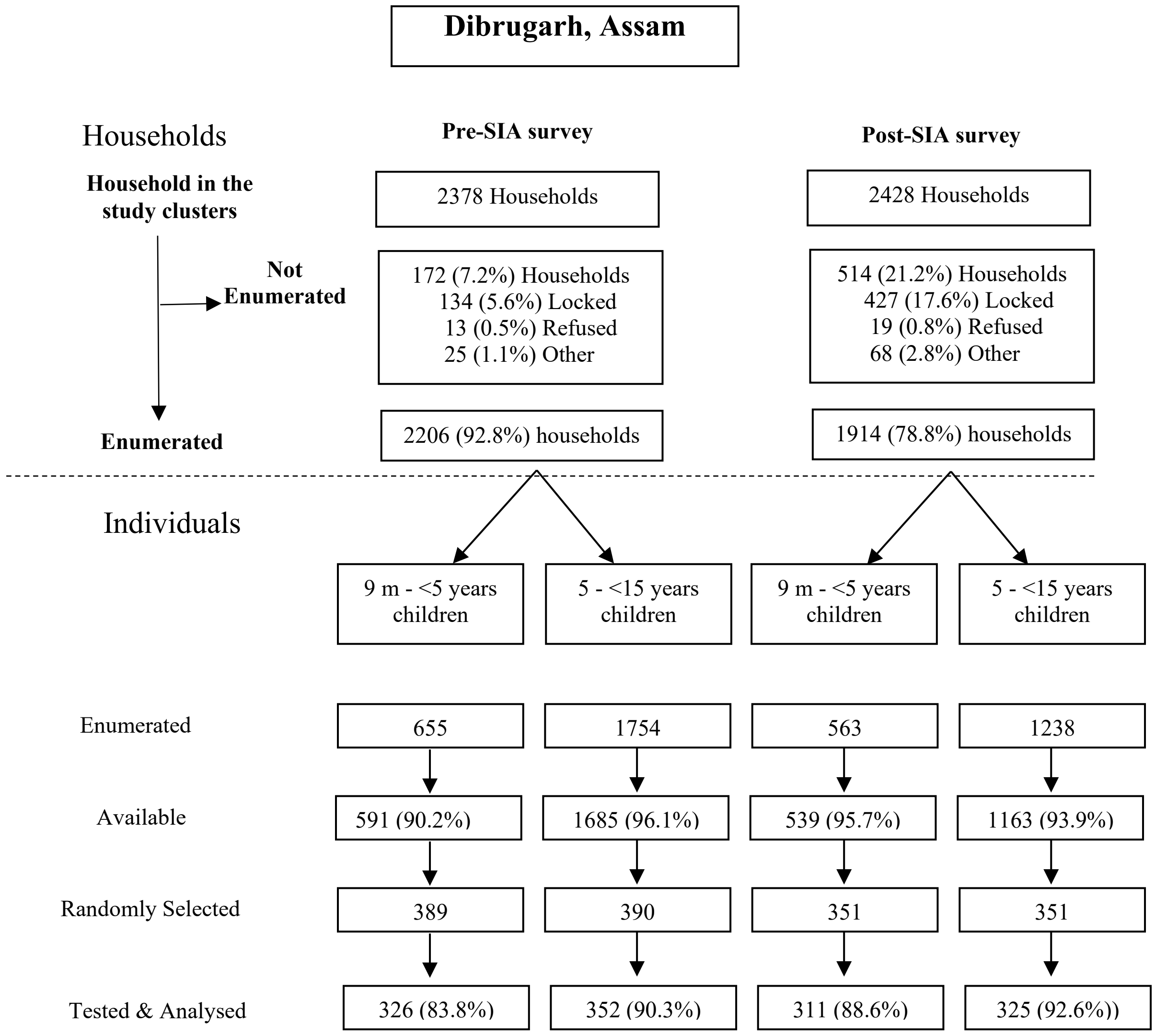

Enumerated: Visited all households in the cluster and collect identification details (name, date of birth/age, gender) **Available:** Children are available for the next three day**s Randomly selected:** Automated selection of children from enumeration data using android application. **Enrolled:** Data collected after obtaining consent/assent **Tested and analysed:** Children with adequate sample volume tested and analysed in the final dataset

Figure 2-C: Flowchart describing the enrolment of participants in the pre and post-SIA serosurveys by district

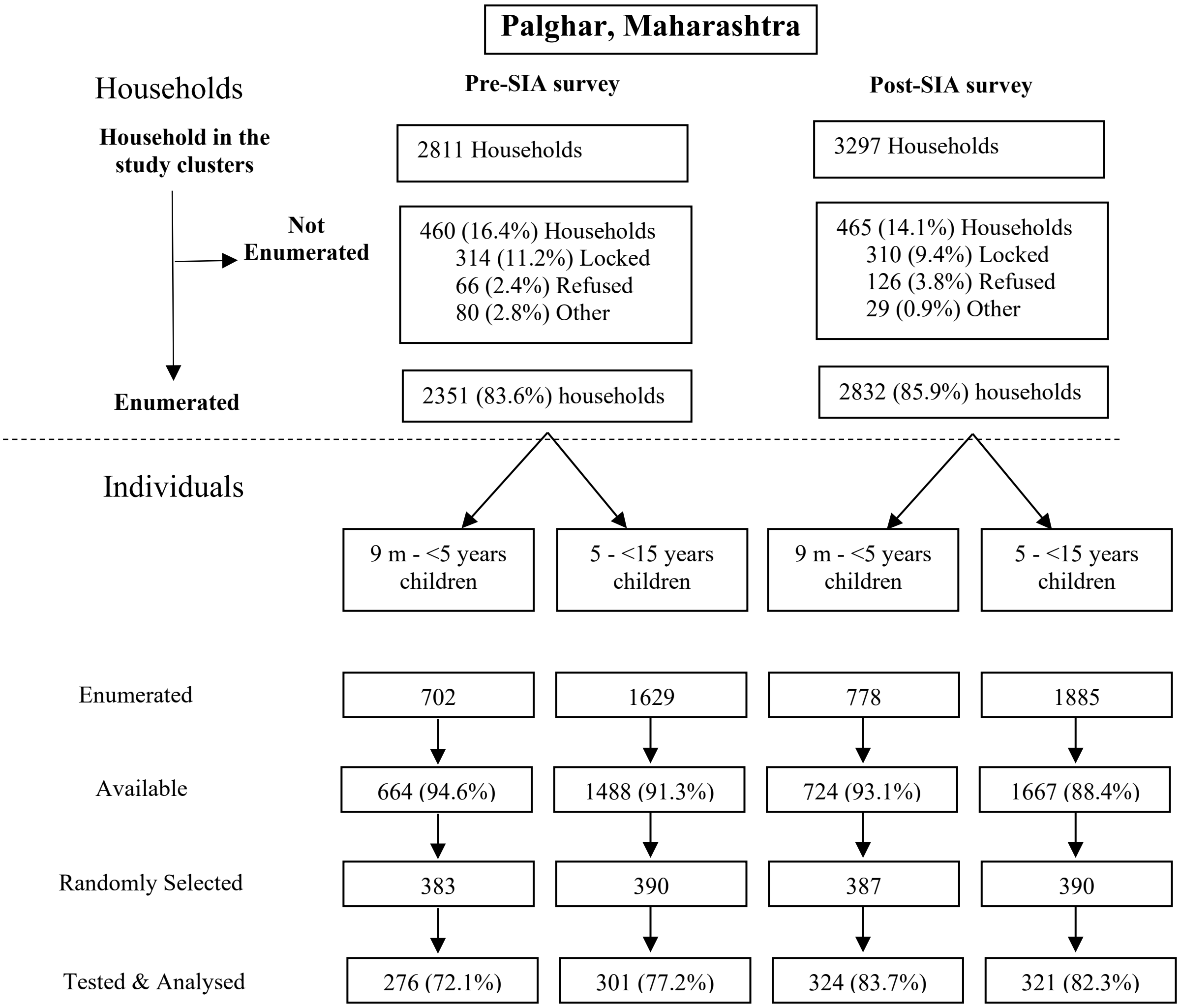

Enumerated: Visited all households in the cluster and collect identification details (name, date of birth/age, gender) **Available:** Children are available for the next three days **Randomly selected:** Automated selection of children from enumeration data using android application. **Enrolled:** Data collected after obtaining consent/assent **Tested and analysed:** Children with adequate sample volume tested and analysed in the final dataset

Figure 2-D: Flowchart describing the enrolment of participants in the pre and post-SIA serosurveys by district

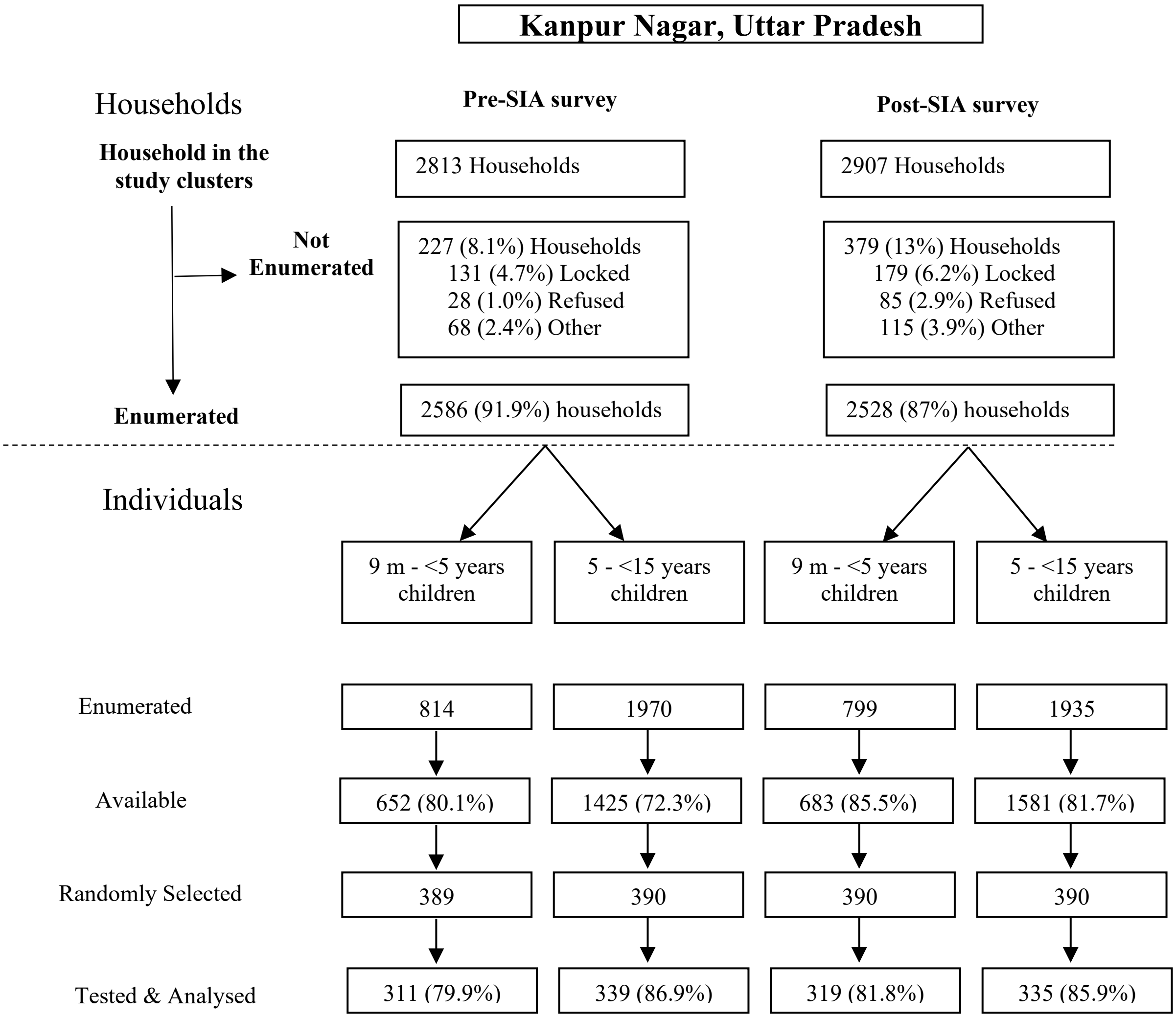

Enumerated: Visited all households in the cluster and collect identification details (name, date of birth/age, gender) **Available:** Children are available for the next three days **Randomly selected:** Automated selection of children from enumeration data using android application. **Enrolled:** Data collected after obtaining consent/assent **Tested and analysed:** Children with adequate sample volume tested and analysed in the final dataset

6. Table 2: Characteristics of participants among children aged 9 months -<5 years by district

Characteristics	Hoshiarpur, Punjab			Dibrugarh, Assam			Palghar, Maharashtra			Kanpur Nagar, Uttar Pradesh		
	**Pre-SIA (n=321)**	**Post-SIA (n=339)**	**P value#**	**Pre-SIA (n=326)**	**Post-SIA (n=311)**	**P Value#**	**Pre-SIA (n=276)**	**Post-SIA (n=324)**	**P value#**	**Pre-SIA (n=311)**	**Post-SIA (n=319)**	**P value#**
**Female sex**	170 (53.0)	166 (49.0)	0.3	174 (53.4)	147 (47.3)	0.12	132 (47.8)	171 (52.8)	0.23	160 (51.4)	150 (47.0)	0.27
**Religion**	n = 307	n = 335	0.07	n = 324	n = 311	0.01	n = 273	n = 324	0.07	n = 311	n = 319	0.07
Hindu	220 (71.7)	231 (69.0)		301 (92.9)	294 (94.5)		255 (93.4)	297 (91.7)		272 (87.5)	293 (91.8)	
Muslim / Christian	12 (3.9)	5 (1.5)		14 (4.3)	17 (5.5)		17 (6.2)	18 (5.6)		39 (12.5)	26 (8.2)	
Sikhs / Buddhist / Jain	75 (24.4)	99 (29.6)		9 (2.8)	0 (0.0)		1 (0.4)	9 (2.8)		0 (0.0)	0 (0.0)	
**Caste**	n = 305	n = 335	0.09	n = 321	n = 301	0.35	n = 267	n = 321	0.07	n = 310	n =319	0.87
General/ Other Backward Class	187 (61.3)	238 (71.0)		264 (82.2)	256 (85.0)		119 (44.6)	179 (55.8)		211 (68.1)	219 (68.7)	
Scheduled Caste / Scheduled Tribe	118 (38.7)	97 (29.0)		57 (17.8)	45 (15.0)		148 (55.4)	142 (44.2)		99 (31.9)	100 (31.3)	
**Mother's Occupation**	n = 321	n = 337	<0.001	n = 325	n = 307	<0.001	n = 275	n = 323	<0.001	n = 309	n = 318	0.08
Employed	20 (6.2)	48 (14.2)		115 (35.4)	58 (19.0)		14 (5.1)	141 (43.6)		13 (4.2)	24 (7.6)	
Home maker	301 (93.8)	289 (85.8)		210 (64.6)	249 (81.1)		261 (94.9)	182 (56.3)		296 (95.8)	294 (92.4)	
**Mother's education**	n = 321	n = 338	0.004	n = 323	n = 307	<0.001	n = 275	n = 324	<0.001	n = 309	n = 318	0.14
Graduate and above	48 (15.0)	72 (21.3)		17 (5.3)	23 (7.5)		21 (7.6)	48 (14.8)		62 (20.1)	77 (24.2)	
11-12 years (higher secondary)	70 (21.8)	90 (26.6)		27 (8.4)	26 (8.5)		12 (4.4)	33 (10.2)		19 (6.1)	32 (10.1)	
6-10 years (middle/high school)	128 (39.9)	131 (38.8)		170 (52.6)	122 (39.7)		126 (45.8)	136 (42.0)		114 (36.9)	114 (35.8)	
≤ 5 years (primary school)	43 (13.4)	22 (6.5)		31 (9.6)	112 (36.5)		52 (18.9)	35 (10.8)		46 (14.9)	42 (13.2)	
Illiterate	32 (10.0)	23 (6.8)		78 (24.1)	24 (7.8)		64 (23.3)	72 (22.2)		68 (22.0)	53 (16.7)	
**Type of house***	n = 309	n = 335	0.001	n = 324	n = 311	<0.001	n = 273	n = 324	0.1	n = 311	n = 319	0.006
Kutcha	11 (3.6)	16 (4.8)		177 (54.6)	224 (72.0)		45 (16.5)	64 (19.8)		19 (6.1)	39 (12.2)	
Pucca	250 (80.9)	298 (89.0)		88 (27.2)	47 (15.1)		106 (38.8)	143 (44.1)		162 (52.1)	176 (55.2)	
Semi-pucca	48 (15.5)	21 (6.3)		59 (18.2)	40 (12.9)		122 (44.7)	117 (36.1)		130 (41.8)	104 (32.6)	

Continued…..Table 2: Characteristics of participants among children aged 9 months -<5 years by district

Characteristics	Hoshiarpur, Punjab			Dibrugarh, Assam			Palghar, Maharashtra			Kanpur Nagar, Uttar Pradesh		
	Pre-SIA (n=321)	Post-SIA (n=339)	P value#	Pre-SIA (n=326)	Post-SIA (n=311)	P value#	Pre-SIA (n=276)	Post-SIA (n=324)	P value#	Pre-SIA (n=311)	Post-SIA (n=319)	P value#
**Type of Residence**	n = 321	n = 339	<0.001	n = 326	n = 311	0.004	n = 276	n = 324	<0.001	n = 311	n = 319	<0.001
Rural	263 (81.9)	225 (66.4)		264 (81.0)	244 (78.5)		162 (58.7)	158 (48.8)		94 (30.2)	114 (35.7)	
Urban Slum	10 (3.1)	11 (3.2)		45 (13.8)	30 (9.6)		30 (10.9)	80 (24.7)		83 (26.7)	116 (36.4)	
Urban Non-Slum	48 (15.0)	103 (30.4)		17 (5.2)	37 (11.9)		84 (30.4)	86 (26.5)		134 (43.1)	89 (27.9)	
**Toilet**	n = 309	n = 335	<0.001	n = 324	n = 311	0.003	n = 273	n = 324	0.27	n = 311	n = 319	<0.001
Own toilet	250 (80.9)	316 (94.3)		286 (88.3)	297 (95.5)		203 (74.4)	259 (79.9)		205 (65.9)	222 (69.6)	
Common toilet/Public toilet	12 (3.9)	2 (0.6)		37 (11.4)	14 (4.5)		30 (11.0)	27 (8.3)		23 (7.4)	61 (19.1)	
No facilities/uses open space	47 (15.2)	17 (5.1)		1 (0.3)	0 (0.0)		40 (14.7)	38 (11.7)		83 (26.7)	36 (11.3)	
**Health seeking behaviour for vaccination**	n = 309	n = 334	0.44	n = 324	n = 311	0.93	n = 273	n = 324	0.23	n = 309	n = 312	<0.001
Public	298 (96.4)	318 (95.2)		314 (96.9)	301 (96.8)		255 (93.4)	294 (90.7)		212 (68.6)	280 (89.7)	
Private / Others	11 (3.6)	16 (4.8)		10 (3.1)	10 (3.2)		18 (6.6)	30 (9.3)		97 (31.4)	32 (10.3)	

SIA: Supplementary Immunization Activities #Chi-square test * **Kutcha house:** Houses made from mud, thatch, or other low-quality materials. **Semi-pucca house:** Houses made from high quality walls with thatched roofs **Pucca house:** Houses made with high quality materials throughout, including the floor, roof, and exterior walls

7. Table 3: Characteristics of participants among children aged 5-<15 years by district

Characteristics	Hoshiarpur, Punjab			Dibrugarh, Assam			Palghar, Maharashtra			Kanpur Nagar, Uttar Pradesh		
	Pre-SIA (n=344)	Post-SIA (n=345)	P value#	Pre-SIA (n=352)	Post-SIA (n=325)	P value#	Pre-SIA (n=301)	Post-SIA (n=321)	P value#	Pre-SIA (n=339)	Post-SIA (n=335)	P value#
**Female sex**	163 (47.4)	168 (48.7)	0.73	174 (49.4)	164 (50.5)	0.79	135 (44.9)	163 (50.8)	0.14	166 (49.0)	146 (43.6)	0.16
**Religion**	n = 334	n = 342	0.34	n = 351	n = 322	0.05	n = 301	n = 321	0.28	n = 338	n = 335	0.25
Hindu	228 (68.3)	229 (67.0)		329 (93.7)	302 (93.8)		278 (92.4)	285 (88.8)		298 (88.2)	303 (90.4)	
Muslim / Christian	12 (3.6)	6 (1.8)		14 (4.0)	19 (5.9)		18 (6.0)	26 (8.1)		40 (11.8)	32 (9.6)	
Sikhs / Buddhist / Jain	94 (28.1)	107 (31.3)		8 (2.3)	1 (0.3)		5 (1.7)	10 (3.1)		0 (0.0)	0 (0.0)	
**Caste**	n = 329	n = 342	0.09	n = 350	n = 316	0.64	n = 295	n = 315	0.004	n = 338	n = 335	0.19
General/Other backward class	194 (59.0)	235 (68.7)		297 (84.9)	264 (83.5)		135 (45.8)	181 (57.5)		220 (65.1)	234 (69.9)	
Scheduled Caste / Scheduled Tribe	135 (41.0)	107 (31.3)		53 (15.1)	52 (16.5)		160 (54.2)	134 (42.5)		118 (34.9)	101 (30.1)	
**Mother's Occupation**	n = 338	n = 340	0.98	n = 339	n = 314	<0.001	n = 297	n = 313	<0.001	n = 334	n = 330	0.001
Employed	34 (10.1)	34 (10.0)		154 (45.4)	82 (26.1)		33 (11.1)	123 (39.3)		21 (6.3)	46 (13.9)	
Home maker	304 (89.9)	306 (90.0)		185 (54.6)	232 (73.9)		264 (89.0)	190 (60.7)		313 (93.7)	284 (86.1)	
**Mother's education**	n = 339	n = 339	0.31	n = 338	n = 314	<0.001	n = 297	n =314	0.002	n = 334	n = 330	0.17
Graduate and above	34 (10.0)	40 (11.8)		17 (5.0)	18 (5.7)		34 (11.4)	29 (9.2)		47 (14.1)	44 (13.3)	
11-12 years (higher secondary)	72 (21.2)	79 (23.3)		20 (5.9)	18 (5.7)		2 (0.7)	17 (5.4)		17 (5.1)	32 (9.7)	
6-10 years (middle/high school)	148 (43.7)	158 (46.6)		162 (47.9)	129 (41.1)		112 (37.7)	133 (42.4)		104 (31.1)	100 (30.3)	
≤ 5 years (primary school)	52 (15.3)	37 (10.9)		40 (11.8)	123 (39.2)		59 (19.9)	40 (12.7)		61 (18.3)	66 (20.0)	
Illiterate	33 (9.7)	25 (7.4)		99 (29.3)	26 (8.3)		90 (30.3)	95 (30.3)		105 (31.4)	88 (26.7)	
**Type of house***	n = 334	n = 342	<0.001	n = 351	n = 322	<0.001	n = 301	n = 321	0.36	n = 338	n = 335	0.11
Kutcha	15 (4.5)	10 (2.9)		192 (54.7)	218 (67.7)		54 (17.9)	48 (15.0)		20 (5.9)	26 (7.8)	
Semi-pucca	57 (17.1)	19 (5.6)		51 (14.5)	53 (16.5)		126 (41.9)	127 (39.6)		148 (43.8)	121 (36.1)	
Pucca	262 (78.4)	313 (91.5)		108 (30.8)	51 (15.8)		121 (40.2)	146 (45.5)		170 (50.3)	188 (56.1)	

Continued…..Table 3: Characteristics of participants among children aged 5-<15 years by district

Characteristics	Hoshiarpur, Punjab			Dibrugarh, Assam			Palghar, Maharashtra			Kanpur Nagar, Uttar Pradesh		
	**Pre-SIA (n=344)**	**Post-SIA (n=345)**	**P value#**	**Pre-SIA (n=352)**	**Post-SIA (n=325)**	**P value#**	**Pre-SIA (n=301)**	**Post-SIA (n=321)**	**P value#**	**Pre-SIA (n=339)**	**Post-SIA (n=335)**	**P value#**
**Type of Residence**	n = 344	n = 345	<0.001	n = 352	n = 325	0.005	n = 301	n = 321	<0.001	n = 339	n = 335	0.001
Rural	276 (80.2)	229 (66.4)		277 (78.7)	251 (77.2)		168 (55.8)	156 (48.6)		98 (28.9)	125 (37.3)	
Urban Slum	13 (3.8)	12 (3.5)		55 (15.6)	35 (10.8)		37 (12.3)	81 (25.2)		97 (28.6)	113 (33.7)	
Urban Non-Slum	55 (16.0)	104 (30.1)		20 (5.7)	39 (12.0)		96 (31.9)	84 (26.2)		144 (42.5)	97 (29.0)	
**Toilet**	n = 344	n = 342	<0.001	n = 351	n = 322	<0.001	n = 301	n = 321	0.24	n = 338	n = 335	<0.001
Own toilet (outside or inside house)	277 (82.9)	327 (95.6)		315 (89.7)	319 (99.1)		235 (78.1)	267 (83.2)		230 (68.0)	256 (76.4)	
Shared common toilet/Public toilet	6 (1.8)	1 (0.3)		35 (10.0)	3 (0.9)		35 (11.6)	26 (8.1)		23 (6.8)	46 (13.7)	
No facilities/uses open space	51 (15.3)	14 (4.1)		1 (0.3)	0 (0.0)		31 (10.3)	28 (8.7)		85 (25.1)	33 (9.9)	
**Health seeking behaviour for vaccination**	n = 333	n = 342	0.02	n = 351	n = 320	0.65	n = 301	n = 320	0.02	n = 337	n = 331	<0.001
Public	313 (94.0)	334 (97.7)		333 (94.9)	306 (95.6)		268 (89.0)	301 (94.1)		228 (67.7)	295 (89.1)	
Private / Others	20 (6.0)	8 (2.3)		18 (5.1)	14 (4.4)		33 (11.0)	19 (5.9)		109 (32.3)	36 (10.9)	

SIA: Supplementary Immunization Activities #Chi-square test * **Kutcha house:** Houses made from mud, thatch, or other low-quality materials. **Semi-pucca house:** Houses made from high quality walls with thatched roofs **Pucca house:** Houses made with high quality materials throughout, including the floor, roof, and exterior walls.

8. **Table 4: Weighted seroprevalence (%) of IgG antibodies against measles before and after MR vaccine SIA by selected characteristics among children aged 9 months to < 5 years, 2018-20**

Characteristics	Hoshiarpur, Punjab		Dibrugarh, Assam		Palghar, Maharashtra		Kanpur Nagar, Uttar Pradesh	
	Pre-SIA (n=321)	Post-SIA (n=339)	Pre-SIA (n=326)	Post-SIA (n=311)	Pre-SIA (n=276)	Post-SIA (n=324)	Pre-SIA (n=311)	Post-SIA (n=319)
**Overall**	81.8 (75.5 - 86.8)	91.5* (85.9 - 95.0)	88.5 (84.6 - 91.5)	94.3* (91.1 - 96.4)	83.1 (75.4 - 88.8)	96.0* (91.4 - 98.2)	80.7 (74.1 - 85.9)	80.4 (74.1 - 85.6)
**Sex**								
Female	81.7 (72.9 - 88.1)	93.4 (88.4 - 96.3)	93.7 (88.9 - 96.5)	92.8 (83.1 - 97.1)	90.2 (79.0 - 95.8)	95.3 (90.9 - 97.6)	83.1 (75.0 - 89.0)	83.0 (73.1 - 89.8)
Male	83.6 (75.8 - 89.3)	91.2 (81.3 - 96.2)	85.5 (79.0 - 90.3)	97.6 (93.7 - 99.1)	79.9 (72.5 - 85.6)	96.1 (86.4 - 99.0)	79.7 (68.6 - 87.6)	78.8 (71.6 - 84.6)
**Locality**								
Rural	83.4 (76.7 - 88.5)	92.4 (88.2 - 95.3)	90.9 (86.8 - 93.8)	96.0 (90.5 - 98.3)	87.3 (77.3 - 93.3)	94.3 (89.4 - 97.0)	85.1 (76.4 - 91.0)	84.8 (73.8 - 91.7)
Urban Slum	60.0 (29.8 - 84.2)	72.8 (41.5 - 91.0)	84.4 (70.8 - 92.4)	86.7 (69.4 - 94.9)	80.0 (62.0 - 90.7)	93.7 (85.8 - 97.4)	75.6 (62.5 - 85.1)	75.9 (66.9 - 83.1)
Urban non-slum	83.3 (70.1 - 91.4)	91.0 (75.6 - 97.0)	89.6 (49.8 - 98.7)	97.3 (83.2 - 99.6)	81.0 (69.6 - 88.8)	98.8 (92.2 - 99.8)	81.1 (69.7 - 88.9)	79.8 (70.2 - 86.9)

MR: Measles and Rubella SIA: Supplementary Immunization Activities * Significant increase in post-SIA seroprevalence compared to pre-SIA (p<0.05)

9. Table 5: Seropositivity against measles by number of MCV doses received among children aged 9 months to <5 years

		Total	Measles Positives (n)	%
	No. of MCV dose received			
**Pre-SIA**	0	210	143	68.1
	1	349	298	85.4
	2	673	589	87.5
**Post- SIA**	0	42	26	61.9
	1	175	146	83.4
	2	300	267	89.0
	3	776	723	93.2

10. **Table 6: Weighted seroprevalence (%) of IgG antibodies against measles before and after MR vaccine SIA by selected characteristics among children aged 5 to <15 years, 2018-20**

Characteristics	Hoshiarpur, Punjab		Dibrugarh, Assam		Palghar, Maharashtra		Kanpur Nagar, Uttar Pradesh	
	Pre-SIA (n=344)	Post-SIA (n=345)	Pre-SIA (n=352)	Post-SIA (n=325)	Pre-SIA (n=301)	Post-SIA (n=321)	Pre-SIA (n=339)	Post-SIA (n=335)
**Overall**	63.4 (56.6 - 69.7)	88.7* (82.6 - 92.9)	75.4 (71.0 - 79.3)	95.9* (91.9 - 98.0)	74.8 (68.8 - 80.0)	96.5* (92.1 - 98.5)	84.5 (78.1 - 89.4)	93.7* (90.9 - 95.7)
**Sex**								
Female	66.8 (58.2 - 74.5)	88.1 (82.3 - 92.2)	81.6 (75.1 - 86.7)	98.8 (95.3 - 99.7)	85.0 (72.6 - 92.4)	95.1 (90.5 - 97.5)	83.9 (76.0 - 89.5)	98.1 (88.6 - 99.7)
Male	59.7 (52.4 - 66.6)	92.2 (83.2 - 96.5)	69.1 (61.9 - 75.5)	93.2 (88.1 - 96.2)	67.5 (59.8 - 74.5)	94.3 (89.4 - 97.0)	87.9 (79.0 - 93.4)	89.9 (84.8 - 93.5)
**Locality**								
Rural	62.1 (54.1 - 69.5)	90.7 (83.2 - 95.0)	74.4 (68.7 - 79.4)	96.0 (92.8 - 97.8)	69.0 (61.7 - 75.6)	94.2 (89.3 - 97.0)	73.7 (63.2 - 82.1)	92.8 (86.7 - 96.2)
Urban Slum	53.9 (28.2 - 77.6)	91.7 (58.7 - 98.8)	78.9 (63.0 - 89.1)	97.1 (82.3 - 99.6)	78.4 (62.4 - 88.8)	95.1 (87.6 - 98.1)	86.4 (75.3 - 93.0)	99.5 (66.3 - 100.0)
Urban non-slum	70.9 (57.7 - 81.3)	90.3 (76.7 - 96.3)	80.0 (57.2 - 92.3)	94.9 (81.7 - 98.7)	77.1 (67.6 - 84.4)	96.1 (82.4 - 99.2)	90.3 (84.2 - 94.2)	88.7 (80.7 - 93.6)

MR: Measles and Rubella SIA: Supplementary Immunization Activities * Significant increase in post-SIA seroprevalence compared to pre-SIA ($p<0.05$)

11. **Table 7: Weighted seroprevalence (%) of IgG antibodies against rubella before and after MR vaccine SIA by selected characteristics among children aged 9 months to < 5 years, 2018-20**

**Characteristics**	**Hoshiarpur, Punjab**		**Dibrugarh, Assam**		**Palghar, Maharashtra**		**Kanpur Nagar, Uttar Pradesh**	
	**Pre-SIA (n=321)**	**Post-SIA (n=339)**	**Pre-SIA (n=326)**	**Post-SIA (n=311)**	**Pre-SIA (n=276)**	**Post-SIA (n=324)**	**Pre-SIA (n=311)**	**Post-SIA (n=319)**
**Overall**	13.0 (8.7 - 19.1)	87.2* (82.7 - 90.7)	10.6 (6.2 - 17.6)	96.5* (91.4 - 98.6)	21.9 (14.5 - 31.7)	94.6* (86.6 - 97.9)	13.9 (8.3 - 22.6)	73.3* (67.5 - 78.4)
**Sex**								
Female	11.9 (5.9 - 22.6)	90.3 (82.3 - 95.0)	11.5 (6.6 - 19.1)	93.5 (83.5 - 97.6)	24.8 (16.5 - 35.6)	95.4 (87.4 - 98.4)	15.8 (9.8 - 24.5)	70.2 (60.7 - 78.3)
Male	13.0 (7.2 - 22.3)	86.3 (79.2 - 91.3)	9.6 (4.6 - 19.1)	96.3 (88.5 - 98.9)	16.5 (8.5 - 29.5)	96.7 (87.1 - 99.2)	15.8 (9.2 - 25.9)	78.1 (68.6 - 85.3)
**Locality**								
Rural	16.0 (11.3 - 22.1)	89.2 (83.2 - 93.3)	8.3 (4.4 - 14.9)	94.9 (88.7 - 97.8)	13.4 (7.1 - 23.8)	98.7 (95.1 - 99.7)	12.8 (7.4 - 21.2)	79.8 (71.5 - 86.2)
Urban Slum	40.0 (15.8 - 70.2)	81.8 (49.3 - 95.4)	22.2 (12.4 - 36.6)	89.3 (56.7 - 98.2)	46.7 (29.9 - 64.2)	90.0 (81.3 - 94.9)	13.7 (4.0 - 37.7)	70.6 (56.3 - 81.7)
Urban non-slum	5.4 (0.9 - 25.6)	86.4 (78.3 - 91.8)	11.7 (2.1 - 44.9)	97.3 (83.2 - 99.6)	27.5 (16.3 - 42.4)	96.0 (72.3 - 99.6)	18.7 (11.7 - 28.7)	69.7 (59.4 - 78.3)

MR: Measles and Rubella SIA: Supplementary Immunization Activities * Significant increase in post-SIA seroprevalence compared to pre-SIA ($p<0.05$)

12. **Table 8: Weighted seroprevalence (%) of IgG antibodies against rubella before and after MR vaccine SIA by selected characteristics among children aged 5 to <15 years, 2018-20**

Characteristics	Hoshiarpur, Punjab		Dibrugarh, Assam		Palghar, Maharashtra		Kanpur Nagar, Uttar Pradesh	
	**Pre-SIA (n=344)**	**Post-SIA (n=345)**	**Pre-SIA (n=352)**	**Post-SIA (n=325)**	**Pre-SIA (n=301)**	**Post-SIA (n=321)**	**Pre-SIA (n=339)**	**Post-SIA (n=335)**
**Overall**	62.2 (55.0 - 68.8)	97.0 (93.0 - 98.7)*	48.7 (41.1 - 56.5)	98.2 (94.5 - 99.4)*	66.5 (58.8 - 73.3)	98.2 (94.8 - 99.4)*	66.8 (61.2 - 71.9)	92.0 (84.9 - 95.9)*
**Sex**								
Female	65.5 (54.5 - 75.0)	95.8 (91.5 - 98.0)	50.4 (40.6 - 60.3)	98.0 (89.9 - 99.6)	67.0 (55.5 - 76.7)	98.8 (95.2 - 99.7)	64.5 (56.9 - 71.4)	91.1 (85.3 - 94.8)
Male	61.3 (54.0 - 68.1)	95.9 (87.7 - 98.7)	49.1 (37.6 - 60.8)	96.0 (86.4 - 98.9)	65.7 (58.1 - 72.5)	97.5 (93.5 - 99.0)	73.7 (64.6 - 81.2)	92.0 (83.1 - 96.4)
**Locality**								
Rural	65.2 (57.3 - 72.4)	97.5 (91.1 - 99.3)	47.4 (37.6 - 57.3)	98.3 (92.4 - 99.6)	59.9 (48.9 - 70.0)	98.1 (94.2 - 99.4)	68.4 (58.5 - 76.8)	94.2 (82.3 - 98.3)
Urban Slum	69.2 (40.9 - 88.0)	NA	50.9 (37.9 - 63.8)	97.1 (82.3 - 99.6)	70.3 (53.9 - 82.7)	98.8 (91.8 - 99.8)	68.3 (53.8 - 79.9)	92.9 (82.3 - 97.3)
Urban non-slum	49.0 (34.0 - 64.2)	93.3 (86.6 - 96.8)	65.0 (42.5 - 82.3)	94.9 (81.7 - 98.7)	74.0 (64.3 - 81.8)	97.6 (91.0 - 99.4)	69.4 (61.5 - 76.4)	85.6 (77.1 - 91.3)

MR: Measles and Rubella SIA: Supplementary Immunization Activities Not applicable since no urban slum clusters surveyed * Significant increase in post-SIA seroprevalence compared to pre-SIA (p<0.05)

13. Table 9: Number (%) of sera samples with equivocal results

	Pre-SIA serosurvey n %	Post-SIA serosurvey n %
**Measles**		
9 months-<5 years	13% (160/1234)	8% (103/1293)
5-<15 years	14% (187/1336)	6% (80/1326)
**Rubella**		
9 months-<5 years	0.1% (1/1234)	0% (0/1293)
5-<15 years	0.6% (8/1336)	0% (0/1326)

14. **Table 10: Weighted overall seroprevalence (%) of IgG antibodies against measles and rubella before and after MR vaccine SIA among considering equivocal samples as test negative**

Seroprevalence	Hoshiarpur, Punjab		Dibrugarh, Assam		Palghar, Maharashtra		Kanpur Nagar, Uttar Pradesh	
	Pre-SIA	Post-SIA	Pre-SIA	Post-SIA	Pre-SIA	Post-SIA	Pre-SIA	Post-SIA
Overall measles seroprevalence (9 months to <5 years)	63.4 (57.4 - 69.1)	83.6 (77.2 - 88.5)	71.8 (67.5 - 75.7)	90.7 (85.6 - 94.1)	74.9 (66.7 - 81.7)	88.0 (82.4 - 92.0)	68.2 (60.0 - 75.4)	68.6 (61.4 - 75.0)
Overall measles seroprevalence (5 to <15 years)	44.4 (36.3 - 52.8)	82.9 (76.3 - 88.0)	55.5 (48.6 - 62.3)	91.6 (85.7 - 95.2)	63.1 (56.1 - 69.6)	91.0 (86.1 - 94.3)	80.9 (73.2 - 86.7)	87.0 (82.3 - 90.6)
Overall rubella seroprevalence (9 months to <5 years)	13.0 (8.7 - 19.1)	87.2 (82.7 - 90.7)	10.6 (6.2 - 17.6)	96.5 (91.4 - 98.6)	21.8 (14.6 - 31.2)	94.6 (86.6 - 97.9)	13.9 (8.3 - 22.6)	73.3 (67.5 - 78.4)
Overall rubella seroprevalence (5 to <15 years)	62.2 (55.0 - 68.8)	97.0 (93.0 - 98.7)	48.7 (41.1 - 56.5)	98.2 (94.5 - 99.4)	64.8 (57.1 - 71.8)	98.2 (94.8 - 99.4)	65.6 (59.9 - 71.0)	92.0 (84.9 - 95.9)

15. Table 11: Factors associated with seronegative status against measles or rubella among children aged 9 months to <5 years in post-SIA survey, 2018-2020

	Measles				Rubella			
Characteristics	N (% seronegative)	P-value[1]	Adjusted Odds ratio (95% CI)	p value[2]	N (% seronegative)	P-value[3]	Adjusted Odds ratio (95% CI)	P-value[4]
**Sex**								
Female	634 (9.6)	0.551	0.91 (0.62-1.35)	0.651	634 (14.4)	0.208	1.25 (0.87-1.82)	0.233
Male	659 (10.6)		REF		659 (12.0)		REF	
**Religion**								
Hindu	1115 (9.5)	0.009	REF		1115 (12.6)	<0.0001	REF	
Muslim / Christian	66 (21.2)		1.64 (0.78-3.47)	0.192	66 (31.8)		2.95 (1.45-5.99)	0.003
Sikhs / Buddhist / Jain	108 (9.3)		1.45 (0.65-3.22)	0.36	108 (8.3)		0.74 (0.33-1.69)	0.476
**Caste**								
General / Other Backward Class / None	892 (9.4)	0.266	-	-	892 (13.7)	0.28	-	
Scheduled Caste / Scheduled Tribe	384 (11.5)		-	-	384 (11.5)		-	
**Mother's Occupation**								
Employed	271 (8.9)	0.466	-	-	271 (9.2)	0.034	1.39 (0.78-2.45)	0.261
Home maker / Unemployed	1014 (10.4)		-	-	1014 (14.1)		REF	
**Mother's education**								
Prof/honors/graduate	220 (7.7)	0.024	REF		220 (10.9)	0.001	REF	
Senior/higher secondary	181 (7.7)		1.24 (0.58-2.68)	0.581	181 (6.6)		0.65 (0.29-1.45)	0.29
High/Middle School	503 (9.7)		1.54 (0.83-2.85)	0.174	503 (12.7)		1.58 (0.87-2.87)	0.133
Primary	211 (10.0)		1.56 (0.73-3.35)	0.255	211 (15.6)		2.51 (1.23-5.13)	0.012
Illiterate	172 (16.9)		1.49 (0.67-3.33)	0.333	172 (20.9)		2.12 (0.97-4.65)	0.06
**Type of house**								
Kutcha	343 (9.3)	0.695	-	-	343 (11.1)	0.033	1.33 (0.63-2.81)	0.458
Semi-pucca	282 (11.3)		-	-	282 (17.7)		1.46 (0.84-2.52)	0.177
Pucca	664 (9.9)		-	-	664 (12.3)		REF	

[1&3] – chi square test; [2]adjusted for sex, religion, mother's education, type of residence, toilet, number of vaccine doses received, and district; [4]adjusted for sex, religion, mother's occupation/education, type of house & residence, toilet, number of vaccine doses received, MR campaign dose coverage and district

Continued Table 11: Factors associated with seronegative status against measles or rubella among children aged 9 months to <5 years in post-SIA survey, 2018-20

	Measles				Rubella			
**Characteristics**	**N (% seronegative)**	**P-value**	**Adjusted Odds ratio (95% CI)**	**p value**	**N (% seronegative)**	**P-value**	**Adjusted Odds ratio (95% CI)**	**P-value**
**Type of Residence**								
Rural	741 (7.7)	<0.0001	REF		741 (9.4)	<0.0001	REF	
Urban Slum	237 (16.9)		1.58 (0.92 - 2.72)	0.101	237 (21.1)		1.26 (0.73 - 2.19)	0.407
Urban Non-Slum	315 (10.8)		1.70 (1.01 - 2.87)	0.046	315 (15.9)		1.96 (1.18 - 3.25)	0.009
**Toilet**								
Own toilet (outside or inside house)	1094 (8.0)	<0.0001	REF		1094 (10.8)	<0.0001	REF	
Shared common toilet/Public toilet	104 (17.3)		1.25 (0.63-2.45)	0.525	104 (26.9)		1.23 (0.64-2.35)	0.542
No facilities/uses open space	91 (26.4)		3.10 (1.57-6.11)	0.001	91 (26.4)		1.67 (0.78-3.61)	0.189
**Health seeking behaviour vaccination**								
Public	1193 (9.7)	0.4	-		1193 (13.2)	0.153	-	
Private / Others	88 (12.5)		-		88 (8.0)		-	
**Total number of measles containing doses received from either campaign or routine, by card or recall**								
0 Dose	42 (38.1)	<0.0001	4.45 (2.00 - 9.87)	<0.0001	-	-	-	-
1 Dose	175 (16.6)		2.97 (1.71 - 5.15)	<0.0001	-	-	-	-
2 Doses	300 (11.0)		1.51 (0.93 - 2.44)	0.096	-	-	-	-
3 Doses	776 (6.8)		REF		-	-	-	-
**MR Campaign Coverage**								
MR Campaign dose documented or by recall	-		-		1097 (7.9)	<0.0001	REF	
No evidence of MR campaign dose by either card or recall	-		-		196 (42.3)		6.77 (4.52 - 10.14)	<0.0001
**District**								
Dibrugarh	311 (5.5)	<0.0001	REF		311 (7.7)	<0.0001	REF	
Hoshiarpur	339 (10.0)		1.81 (0.88 - 3.69)	0.106	339 (12.4)		2.48 (1.15-5.37)	0.021
Kanpur Nagar	319 (20.4)		3.59 (1.86 - 6.93)	<0.0001	319 (27.0)		2.98 (1.49-5.97)	0.002
Palghar	324 (4.6)		0.77 (0.35 - 1.67)	0.505	324 (5.6)		0.71 (0.32-1.58)	0.403

16. Table 12: Factors associated with seronegative status against measles or rubella among children aged 5 to <15 years in post-SIA survey, 2018-2020

	Measles				Rubella			
Characteristics	N (% seronegative)	P-value[1]	Adjusted Odds ratio (95% CI)	p value[2]	N (% seronegative)	P-value[3]	Adjusted Odds ratio (95% CI)	P-value[4]
**Sex**								
Female	641 (5.8)	0.037	0.64 (0.41-0.98)	0.042	641 (4.5)	0.234	0.84 (0.50 - 1.43)	0.526
Male	685 (8.8)		REF		685 (6.0)		REF	
**Religion**								
Hindu	1119 (7.3)	0.214			1119 (5.1)	0.725		
Muslim / Christian	83 (3.6)				83 (4.8)			
Sikhs / Buddhist / Jain	118 (10.2)				118 (6.8)			
**Caste**								
General / Other Backward Class / None	914 (6.8)	0.24			914 (5.7)	0.224		
Scheduled Caste / Scheduled Tribe	394 (8.6)				394 (4.1)			
**Mother's Occupation**								
Employed	285 (8.1)	0.626			285 (5.6)	0.75		
Home maker / Unemployed	1012 (7.2)				1012 (5.1)			
**Mother's education**								
Prof/honors/graduate	131 (7.6)	0.839			131 (5.3)	0.942		
Senior/higher secondary	146 (7.5)				146 (5.5)			
High/Middle School	520 (7.9)				520 (4.6)			
Primary	266 (5.6)				266 (5.6)			
Illiterate	234 (7.7)				234 (6.0)			
**Type of house**								
Kutcha	302 (5.0)	0.008	0.97 (0.44-2.11)	0.93	302 (5.0)	0.928		
Semi-pucca	320 (5.0)		0.71 (0.38-1.34)	0.286	320 (5.6)			
Pucca	698 (9.5)		REF		698 (5.2)			

[1&3] – chi square test; [2]adjusted for sex, type of house, residence, MR campaign coverage, and district; [4]adjusted for sex, type of residence, MR campaign dose coverage and district

Continued Table 12: Factors associated with seronegative status against measles or rubella among children aged 5 to <15 years in post-SIA survey, 2018-2020

	Measles				Rubella			
**Characteristics**	**N (% seronegative)**	**P-value**	**Adjusted Odds ratio (95% CI)**	**p value**	**N (% seronegative)**	**P-value**	**Adjusted Odds ratio (95% CI)**	**P-value**
**Type of Residence**								
Rural	761 (7.2)	0.153	REF		761 (4.3)	0.073	REF	
Urban Slum	241 (5.0)		0.63 (0.31 - 1.27)	0.197	241 (5.0)		0.49 (0.22 - 1.07)	0.073
Urban Non-Slum	324 (9.3)		1.05 (0.62 - 1.76)	0.864	324 (7.7)		1.31 (0.73 - 2.37)	0.366
**Toilet**								
Own toilet (outside or inside house)	1169 (7.4)	0.936			1169 (5.1)	0.846		
Shared common toilet/Public toilet	76 (6.6)				76 (5.3)			
No facilities/uses open space	75 (6.7)				75 (6.7)			
**Health seeking behaviour vaccination**								
Public	1236 (7.2)	0.536			1236 (5.2)	0.995		
Private / Others	77 (9.1)				77 (5.2)			
**MR Campaign Coverage**								
MR Campaign dose documented or by recall	1149 (6.1)	<0.0001	REF		1149 (2.5)	<0.0001	REF	
No evidence of MR campaign dose by either card or recall	176 (15.3)		2.74 (1.66 - 4.52)	<0.0001	176 (23.3)		10.7 (6.21 - 18.3)	<0.0001
**District**								
Dibrugarh	325 (4.0)		REF		325 (4.3)	<0.0001	REF	
Hoshiarpur	345 (11.9)		2.81 (1.23 - 6.42)	0.014	345 (4.3)		0.72 (0.33 - 1.58)	0.416
Kanpur Nagar	335 (7.8)		1.84 (0.81 - 4.18)	0.147	335 (10.4)		1.79 (0.89 - 3.62)	0.105
Palghar	321 (5.3)		1.59 (0.70 - 3.65)	0.271	321 (1.9)		0.52 (0.19 - 1.42)	0.205

17. Table 13: Factors associated with seronegative status against measles or rubella among children aged 9 months to <5 years in pre-SIA survey, 2018-2020

	Measles				Rubella			
**Characteristics**	**N (% seronegative)**	**p value**	**Adjusted Odds ratio (95% CI)**	**p.value**	**N (% seronegative)**	**p value**	**Adjusted Odds ratio (95% CI)**	**p value**
**Sex**								
Female	636(14.3)	0.036	0.63(0.46 - 0.87)	0.005	636(82.2)	0.520		
Male	598(18.7)		REF		598(83.6)			
**Religion**								
Hindu	1048(15.3)	0.004	REF		1048(83.8)	0.010	REF	
Muslim / Christian	82(29.3)		2.07(1.20 - 3.58)	0.009	82(70.7)		0.54(0.31 - 0.95)	0.031
Sikhs / Buddhist / Jain	85(18.8)		1.37(0.71 - 2.62)	0.345	85(83.5)		0.94(0.48 - 1.85)	0.857
**Caste**								
General / Other Backward Class / None	781(15.7)	0.366			781(81.9)	0.244		
Scheduled Caste / Scheduled Tribe	422(17.8)				422(84.6)			
**Mother's Occupation**								
Employed	162(13.6)	0.308			162(87.7)	0.081	REF	
Home maker / Unemployed	1068(16.8)				1068(82.1)		0.76(0.43 - 1.34)	0.34
**Mother's education**								
Prof/honors/graduate	148(16.2)	0.922			148(70.9)	<0.001	REF	
Senior/higher secondary	128(14.1)				128(79.7)		1.54(0.84 - 2.81)	0.163
High/Middle School	538(16.5)				538(86.1)		2.75(1.69 - 4.47)	<0.001
Primary	172(15.7)				172(82.6)		2.10(1.17 - 3.79)	0.014
Illiterate	242(17.8)				242(84.7)		2.10(1.19 - 3.72)	0.011
**Type of house**								
Kutcha	252(11.9)	0.089	1.16(0.64 - 2.08)	0.627	252(87.3)	0.010	1.20(0.70 - 2.08)	0.506
Semi-pucca	359(18.1)		1.30(0.83 - 2.04)	0.253	359(85.2)		1.37(0.87 - 2.14)	0.173
Pucca	606(17.3)		REF		606(79.7)		REF	

Continued Table 13: Factors associated with seronegative status against measles or rubella among children aged 9 months to <5 years in pre-SIA survey, 2018-2020

Characteristics	N (% seronegative)	p value	Adjusted Odds ratio (95% CI)	p.value	N (% seronegative)	p value	Adjusted Odds ratio (95% CI)	p value
**Type of Residence**								
Rural	783(14.0)	0.008	REF		783(85.8)	<0.001	REF	
Urban Slum	168(22.6)		1.43(0.87 - 2.37)	0.160	168(73.8)		0.49(0.31 - 0.78)	0.002
Urban Non-Slum	283(19.4)		1.22(0.77 - 1.94)	0.398	283(80.2)		1.12(0.71 - 1.77)	0.628
**Toilet**								
Own toilet (outside or inside house)	944(15.6)	0.179	REF		944(82.6)	0.874		
Shared common toilet/Public toilet	102(22.5)		1.40(0.78 - 2.50)	0.261	102(83.3)			
No facilities/uses open space	171(17.5)		0.89(0.53 - 1.50)	0.664	171(84.2)			
**Health seeking behaviour vaccination**								
Public	1079(15.8)	0.098	REF		1079(84.3)	<0.001	REF	
Private / Others	136(21.3)		1.36(0.82 - 2.27)	0.232	136(71.3)		0.51(0.32 - 0.82)	0.006
**Total number of measles containing doses received from either campaign or routine, by card or recall**								
0 Dose	210(31.9)	<0.001	3.32(2.24 - 4.92)	<0.001	-	-	-	-
1 Dose	349(14.6)		1.38(0.94 - 2.04)	0.101	-	-	-	-
2 Doses	673(12.5)		REF		-	-	-	-
District					-	-	-	-
Dibrugarh	326(10.1)		REF		326(87.4)		REF	
Hoshiarpur	321(18.4)	0.003	2.32(1.28 - 4.21)	0.006	321(84.1)	0.006	0.98(0.57 - 1.71)	0.934
Kanpur Nagar	311(20.6)		1.51(0.83 - 2.77)	0.177	311(82.3)		1.14(0.66 - 1.99)	0.635
Palghar	276(17.0)		1.55(0.89 - 2.69)	0.122	276(76.8)		0.49(0.29 - 0.81)	0.05

18. **Table 14: Factors associated with seronegative status against measles or rubella among children aged 5 years to <15 years in pre-SIA survey, 2018-2020**

Characteristics	N (% seronegative)	p value	Adjusted Odds ratio (95% CI)	p value	N (% seronegative)	p value	Adjusted Odds ratio (95% CI)	p value
**Sex**								
Female	638(22.4)	0.002	0.66(0.51 - 0.86)	0.002	638(39.7)	0.599		
Male	698(29.8)		REF		698(38.3)			
**Religion**								
Hindu	1133(26.3)	0.002	REF		1133(39.6)	0.413		
Muslim / Christian	84(13.1)		0.50(0.24 - 1.00)	0.052	84(34.5)			
Sikhs / Buddhist / Jain	107(35.5)		0.88(0.54 - 1.42)	0.592	107(34.6)			
**Caste**								
General / Other Backward Class / None	846(24.6)	0.084	REF		846(38.1)	0.375		
Scheduled Caste / Scheduled Tribe	466(29.0)		1.03(0.76 - 1.38)	0.872	466(40.6)			
**Mother's Occupation**								
Employed	242(25.6)	0.813			242(38.8)	0.999		
Home maker / Unemployed	1066(26.4)				1066(38.8)			
**Mother’s education**								
Prof/honors/graduate	132(25.0)	0.102	REF		132(31.8)	0.001	REF	
Senior/higher secondary	111(30.6)		0.81(0.44 - 1.51)	0.510	111(27.0)		0.62(0.34 - 1.12)	0.115
High/Middle School	526(29.3)		1.00 (0.61 - 1.63)	0.989	526(45.1)		1.29(0.83 - 2.02)	0.257
Primary	212(22.6)		0.71(0.40 - 1.25)	0.235	212(35.8)		0.95(0.57 - 1.58)	0.842
Illiterate	327(22.3)		0.74(0.44 - 1.27)	0.278	327(37.6)		0.96(0.59 - 1.57)	0.866
**Type of house**								
Kutcha	281(23.1)	0.405			281(46.6)	0.009	1.13(0.78 - 1.63)	0.527
Semi-pucca	382(27.5)				382(38.2)		1.14(0.83 - 1.57)	0.41
Pucca	661(26.8)				661(36.0)		REF	

Continued Table 14: Factors associated with seronegative status against measles or rubella among children aged 5 to <15 years in pre-SIA survey, 2018-2020

	Measles				Rubella			
**Characteristics**	**N (% seronegative)**	**p value**	**Adjusted Odds ratio (95% CI)**	**p value**	**N (% seronegative)**	**p value**	**Adjusted Odds ratio (95% CI)**	**p value**
**Type of Residence**								
Rural	819(31.1)	<0.001	REF		819(41.8)	0.020	REF	
Urban Slum	202(19.8)		0.77(0.51 - 1.17)	0.220	202(36.6)		0.92(0.65 - 1.31)	0.655
Urban Non-Slum	315(17.8)		0.56(0.38 - 0.83)	0.004	315(33.0)		0.99(0.70 - 1.40)	0.956
**Toilet**								
Own toilet (outside or inside house)	1057(26.3)	0.975			1057(38.3)	0.584		
Shared common toilet/Public toilet	99(25.3)				99(43.4)			
No facilities/uses open space	168(26.2)				168(39.9)			
**Health seeking behaviour vaccination**								
Public	1142(27.4)	0.160			1142(40.7)	0.001	REF	
Private / Others	180(18.9)				180(27.8)		0.70(0.47 - 1.04)	0.081
**District**								
Dibrugarh	352(24.7)	<0.001	REF		352(50.6)	<0.001	REF	
Hoshiarpur	344(37.2)		1.82(1.24 - 2.69)	0.002	344(37.8)		0.68(0.47 - 0.97)	0.035
Kanpur Nagar	339(15.9)		0.74(0.48 - 1.14)	0.170	339(31.6)		0.53(0.36 - 0.78)	0.001
Palghar	301(27.2)		1.29(0.86 - 1.93)	0.213	301(34.9)		0.54(0.37 - 0.76)	0.001

19. Table 15. Summary of risk factors associated with measles and rubella seronegativity in pre and post SIA serosurveys

	Pre-SIA	Post-SIA
**Measles (9 months to <5 years)**	Male sex	-
	Muslim/Christian	-
	‘0’ MCV dose	‘0’ or ‘1’ MCV dose
	Hoshiarpur	-
	-	No toilet facilities/uses open space
	-	Kanpur Nagar
**Measles (5 to <15 years)**	Male sex	Female sex
	Muslim/Christian	-
	Hoshiarpur	Hoshiarpur
	-	Non-receipt of SIA dose
**Rubella (9 months to <5 years)**	Mothers Education (high school/primary/Illiterate)	Mothers education (primary)
	Palghar	-
	-	Urban Non-slum
	-	Non-receipt of SIA dose
	-	Muslim/Christian
	-	Kanpur Nagar and Hoshiarpur
**Rubella (5 to <15 years)**	-	Non-receipt of SIA dose

20. Table 16: Children age 12-23 months who have received measles vaccine (%) in the surveyed districts from National Family Health Survey (NFHS)

	Hoshiarpur, Punjab	Dibrugarh, Assam	Palghar, Maharashtra	Kanpur Nagar, Uttar Pradesh
NFHS-4 (2015-2016) -One dose	95.2	92.6	72.3*	74.0
NFHS-5 (2019-2020)- First dose	95.4	97.8	100	78.6
NFHS-5 (2019-2020)- Second dose	29.2	35.1	31.3	32.2

*Earlier Palghar was part of Thane district

21. **Table 17: Comparison of predicted seroprevalence from the MCV coverage estimates and observed seroprevalence estimated during post-SIA serosurvey**

	Hoshiarpur	Dibrugarh	Palghar	Kanpur
**mcv1 (%)**	95.8	98.8	98.9	93.8
**mcv2 (%)**	84.9	71.2	90.7	83.4
**Predicted measles seroprevalence**				
(mcv1*mcv2*ve2) + (mcv1*(1-mcv2)*ve1)[a]	91.0	92.1	94.7	88.9
(ve1*mcv1)+(ve2*mcv2)-(mcv2*ve1*ve2*mcv1)[b]	96.6	94.7	97.9	95.9
**Observed measles prevalence**	91.5<br>(85.9 - 95.0)	94.3<br>(91.1 - 96.4)	96.0<br>(91.4 - 98.2)	80.4<br>(74.1 - 85.6)

ve1=84%; ve2=97%; [a]dependence assumption: receipt of MCV2 is dependent on receipt of MCV1 [b]Independence assumption: Receipt of MCV2 is independent of MCV1 (randomly distributed throughout population)

22. Strengthening the Reporting of Observational Studies in Epidemiology (STROBE) checklist

	Item No	Recommendation	Location in the manuscript
**Title and abstract**	1	(*a*) Indicate the study's design with a commonly used term in the title or the abstract	Line 1-3
		(*b*) Provide in the abstract an informative and balanced summary of what was done and what was found	Line 93-118
**Introduction**			
Background/rationale	2	Explain the scientific background and rationale for the investigation being reported	Line 180-218
Objectives	3	State specific objectives, including any prespecified hypotheses	Line 215-218
**Methods**			
Study design	4	Present key elements of study design early in the paper	Line 223
Setting	5	Describe the setting, locations, and relevant dates, including periods of recruitment, exposure, follow-up, and data collection	Line 223-230
Participants	6	(*a*) Give the eligibility criteria, and the sources and methods of selection of participants	Line 232-241
Variables	7	Clearly define all outcomes, exposures, predictors, potential confounders, and effect modifiers. Give diagnostic criteria, if applicable	Line 255-258, Line 278-282
Data sources/ measurement	8*	For each variable of interest, give sources of data and details of methods of assessment (measurement). Describe comparability of assessment methods if there is more than one group	
Bias	9	Describe any efforts to address potential sources of bias	
Study size	10	Explain how the study size was arrived at	Line 243-245
Quantitative variables	11	Explain how quantitative variables were handled in the analyses. If applicable, describe which groupings were chosen and why	Line 304-311
Statistical methods	12	(*a*) Describe all statistical methods, including those used to control for confounding	Line 304-321
		(*b*) Describe any methods used to examine subgroups and interactions	NA
		(*c*) Explain how missing data were addressed	
		(*d*) If applicable, describe analytical methods taking account of sampling strategy	Line 310-311 appendix p5
		(*e*) Describe any sensitivity analyses	Line 283-284
**Results**			
Participants	13*	(a) Report numbers of individuals at each stage of study—eg numbers potentially eligible,	Line 335-347

		examined for eligibility, confirmed eligible, included in the study, completing follow-up, and analysed	
		(b) Give reasons for non-participation at each stage	Line 335-347
		(c) Consider use of a flow diagram	Figure 1 and Appendix figure 2
Descriptive data	14*	(a) Give characteristics of study participants (eg demographic, clinical, social) and information on exposures and potential confounders	Line 351-359 Table 1 and Tables 2&3
		(b) Indicate number of participants with missing data for each variable of interest	Table 1 and Tables 2&3
Outcome data	15*	Report numbers of outcome events or summary measures	NA
Main results	16	(*a*) Give unadjusted estimates and, if applicable, confounder-adjusted estimates and their precision (eg, 95% confidence interval). Make clear which confounders were adjusted for and why they were included	Line 373-402 Table 2, Appendix tables 4-10
		(*b*) Report category boundaries when continuous variables were categorized	Line 279-281
		(*c*) If relevant, consider translating estimates of relative risk into absolute risk for a meaningful time period	
Other analyses	17	Report other analyses done—eg analyses of subgroups and interactions, and sensitivity analyses	Appendix table 10
**Discussion**			
Key results	18	Summarise key results with reference to study objectives	Line 434-439
Limitations	19	Discuss limitations of the study, taking into account sources of potential bias or imprecision. Discuss both direction and magnitude of any potential bias	Line 514-525
Interpretation	20	Give a cautious overall interpretation of results considering objectives, limitations, multiplicity of analyses, results from similar studies, and other relevant evidence	Line 445-503
Generalisability	21	Discuss the generalisability (external validity) of the study results	Line 514-516
**Other information**			
Funding	22	Give the source of funding and the role of the funders for the present study and, if applicable, for the original study on which the present article is based	Line 325-330

*Give information separately for exposed and unexposed groups.
